# Selective Sirt2 inhibition by ligand-induced rearrangement of the active site

**DOI:** 10.1038/ncomms7263

**Published:** 2015-02-12

**Authors:** Tobias Rumpf, Matthias Schiedel, Berin Karaman, Claudia Roessler, Brian J. North, Attila Lehotzky, Judit Oláh, Kathrin I. Ladwein, Karin Schmidtkunz, Markus Gajer, Martin Pannek, Clemens Steegborn, David A. Sinclair, Stefan Gerhardt, Judit Ovádi, Mike Schutkowski, Wolfgang Sippl, Oliver Einsle, Manfred Jung

**Affiliations:** 1 Institute of Pharmaceutical Sciences, Albert-Ludwigs-University Freiburg, Albertstraße 25, 79104 Freiburg im Breisgau, Germany; 2 Institute for Pharmacy, Martin-Luther-University Halle-Wittenberg, Wolfgang-Langenbeck-Straße 4, 06120 Halle (Saale), Germany; 3 Institute for Biochemistry and Biotechnology, Martin-Luther-University Halle-Wittenberg, Kurt-Mothes-Straße 3, 06120 Halle (Saale) Germany; 4 Department of Genetics, Harvard Medical School, 77 Avenue Louis Pasteur, Boston, Massachusetts 02115, USA; 5 Institute of Enzymology, Research Centre for Natural Sciences, Hungarian Academy of Sciences, Magyar Tudósok körútja 2, H-1117 Budapest, Hungary; 6 Department of Biochemistry, University of Bayreuth, Universitätsstraße 30, 95445 Bayreuth, Germany; 7 Institute for Biochemistry and BIOSS Centre for Biological Signalling Studies, Albert-Ludwigs-University Freiburg im Breisgau, Albertstraße 21, 79104 Freiburg im Breisgau, Germany

## Abstract

Sirtuins are a highly conserved class of NAD^+^-dependent lysine deacylases. The human isotype Sirt2 has been implicated in the pathogenesis of cancer, inflammation and neurodegeneration, which makes the modulation of Sirt2 activity a promising strategy for pharmaceutical intervention. A rational basis for the development of optimized Sirt2 inhibitors is lacking so far. Here we present high-resolution structures of human Sirt2 in complex with highly selective drug-like inhibitors that show a unique inhibitory mechanism. Potency and the unprecedented Sirt2 selectivity are based on a ligand-induced structural rearrangement of the active site unveiling a yet-unexploited binding pocket. Application of the most potent Sirtuin-rearranging ligand, termed SirReal2, leads to tubulin hyperacetylation in HeLa cells and induces destabilization of the checkpoint protein BubR1, consistent with Sirt2 inhibition *in vivo*. Our structural insights into this unique mechanism of selective sirtuin inhibition provide the basis for further inhibitor development and selective tools for sirtuin biology.

## Abstract (web summary)

The involvement of the sirtuin family of lysine deacylases in disease, metabolism and ageing makes them promising pharmaceutical targets. Rumpf *et al.* present structures of human Sirt2 in complex with two highly selective drug-like inhibitors, and show that they act by rearranging the enzyme’s active site.

## Graphical abstract

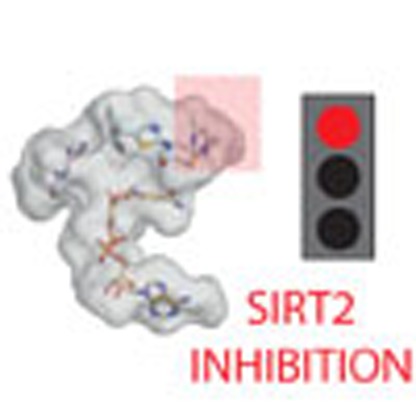

NAD^+^-dependent protein deacylases (sirtuins) constitute a unique class of enzymes that are conserved from bacteria to humans. Initially recognized as protein deacetylases, they were recently shown to catalyse further post-translational modifications such as demyristoylation^1^,^2^, desuccinylation^3^ or ADP-ribosylation^4^. Through a multitude of protein substrates, they are involved in key cellular processes, including metabolic sensing, regulation of mitosis and aging. The human isotype Sirtuin 2 (Sirt2) deacetylates both nuclear and cytoplasmatic proteins and thereby functions as a major cell cycle regulator^5^,^6^, a determinant of myelination^7^, a regulator of autophagy^8^ and a suppressor of brain inflammation^9^. Generally deemed as a tumour suppressor^10^,^11^ in some types of cancer, Sirt2 was also shown to adopt a contrary role by promoting tumorigenesis^12^,^13^. In addition, recent reports link Sirt2 to bacteria-induced reprogramming of host cell gene expression^14^.

Owing to its multiple regulatory roles, Sirt2 has been implicated as a potential drug target to combat cancer^12^,^13^, neurodegeneration^15^,^16^ and inflammation^7^ but other reports question the suitability of Sirt2 as a drug target^17^. The physiological studies of Sirt2 have so far been hampered by the lack of potent and isotype-specific modulators of sirtuin activity.

The biochemistry of sirtuins has been studied extensively in recent years and three-dimensional structures of the catalytic domain of several human isotypes provided insight into different stages of the catalytic cycle^18^,^19^,^20^,^21^,^22^,^23^. Despite a highly conserved amino-acid sequence and a high structural similarity of the catalytic core between the members of the sirtuin family, recent screening campaigns have identified several isotype-selective inhibitors^15^,^24^,^25^,^26^,^27^. But for only a few of them structural information is available (Supplementary Fig. 1a) and a strategy for structure-based optimization of isotype-selective inhibitors remains mostly elusive. In particular, a structure-derived rationale for Sirt2-selective inhibition is also lacking so far. Most recently, the first X-ray structure of Sirt2 in complex with a potent macrocyclic peptidic inhibitor was reported, but this inhibitor lacked the desired isotype selectivity^28^ and, due to its physicochemical properties, might be of limited use for drug discovery. In this work, we present the first crystal structures of Sirt2 in complex with a potent and Sirt2-selective small-molecule inhibitor with drug-like properties. The basis for the high potency and unprecedented isotype selectivity is a ligand-induced structural rearrangement of the active site, exploiting an adjacent binding pocket. Along with kinetic studies, the structures give insight into a unique and isotype-selective inhibition mechanism. The relevance of the observed biochemical activity is further supported by cellular studies.

## Results

### Identification and crystallization of SirReal inhibitors

**Figure 1:**
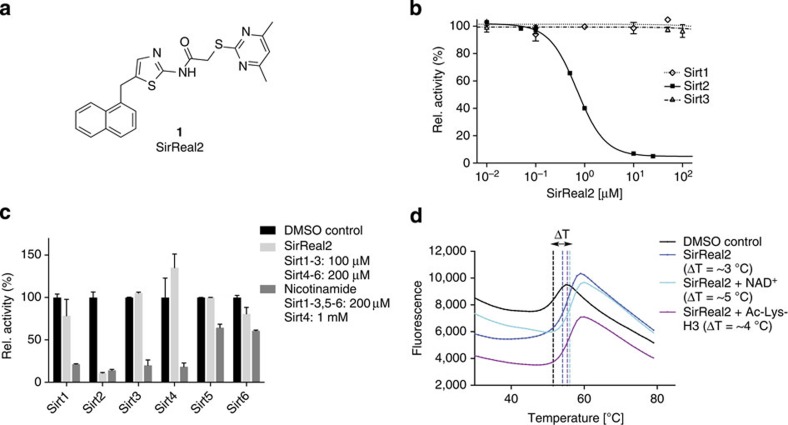
SirReal2 selectively inhibits Sirt2 in a dose-dependent manner. (**a**) Chemical structure of SirReal2 (**1**). (**b**) Representative dose–response curve for Sirt1–3 and SirReal2 using the substrates ZMAL (Z-Lys(Acetyl)-AMC, Sirt1-2) resp. Fluor-de-Lys (Sirt3). Compared with the peptide-HPLC assay, SirReal2 was slightly less potent using ZMAL with an IC_50_ value of 0.4 μM. Data are presented as mean±s.d. (*n*=3). (**c**) *In vitro* inhibition data for SirReal2 (Sirt1–3: 100 μM; Sirt4–6: 200 μM) in an assay using non-labelled acyl-lysine oligopeptide as a substrate (Sirt1–4, acetyl-lysine substrate; Sirt5, succinyl-lysine substrate; Sirt6, myristoyl-lysine substrate). A solution containing DMSO was used as a negative control, a solution with nicotinamide (NCA, 200 μM or 1 mM) was used as a positive control. Only the activity of Sirt2 is substantially reduced in the presence of SirReal2. Data are presented as mean±s.d. (*n*=2) (**d**) Representative thermal stability plots for Sirt2 in the presence of SirReal2 (25 μM) and either the cofactor NAD^+^ (5 mM) or an acetyl-lysine H3 peptide (5 mM). The presence of NAD^+^ or of an acetyl-lysine peptide substrate enhances the stability of the Sirt2–SirReal2 complex (*n*=3). Representative thermal stability plots of Sirt2 in the absence of SirReal2 and in the presence of NAD^+^ or an acetyl-lysine oligopeptide are shown in Supplementary Fig. 1d. Rel., relative.

In search for new sirtuin inhibitors, we screened an internal compound library using an *in vitro* assay^29^ based on a fluorophore-labelled acetyl-lysine derivative for human Sirt1–3. In this screening campaign, a family of aminothiazoles that we have termed Sirtuin-rearranging ligands (SirReals) was discovered as potent, Sirt2-selective inhibitors. Of these, SirReal2 (**1**) showed the most promising inhibitory properties (Fig. 1a,b). AGK2 was used as a reference inhibitor. Under the same assay conditions it is 38-fold less potent with an IC_50_ of 15.4±0.7 μM. The activity of Sirt1 or Sirt3 was not affected at 50 μM. Additional confirmation of Sirt2-selective *in vitro* inhibition and binding by SirReal2 was obtained by using non-labelled peptidic substrates in a high-performance liquid chromatography (HPLC)-based conversion assay (Fig. 1c, Supplementary Fig. 1b) and from thermal stability assays, where the presence of SirReal2 led to increased melting temperatures due to ligand-induced stabilization of the protein (Fig. 1d). SirReal2 only inhibits Sirt2 potently with an IC_50_ value of 140 nM and has very little effect on the activities of Sirt3-5. Only the activity of Sirt1 (22% inhibition at 100 μM) and Sirt6 (19% inhibition at 200 μM) are slightly affected at higher SirReal2 concentrations, making SirReal2 one of the most selective sirtuin inhibitors up to date. However, any attempts to identify a putative-binding site and to rationalize initial structure–activity relationships by docking to available X-ray structures of Sirt2 were not successful. We, therefore, proceeded to determine the structures of Sirt2-inhibitor complexes by protein X-ray crystallography.

For that, we used a truncated form of Sirt2_56–356_ lacking the flexible N- and C termini. To validate the suitability of our expression construct, we also crystallized this truncated form of Sirt2 in the presence of ADP ribose (ADPR) and the physiological inhibitor nicotinamide (NCA, Supplementary Fig. 2, structure termed Sirt2–ADPR–NCA). As the binding mode of these ligands corresponded to other published sirtuin structures in complex with NCA^30^, we concluded that our expression construct was suitable for the investigation of Sirt2–ligand interactions. Further thermal stability experiments indicated an additional stabilization of the Sirt2–SirReal2 complex in the presence of either NAD^+^ or a peptidic acetyl-lysine substrate (Fig. 1d). These findings were the key to a successful crystallization of Sirt2 in complex with SirReal2 that was only achieved in the presence of either substrate or cosubstrate.

### Overall structure of Sirt2–SirReal2 complexes

**Figure 2:**
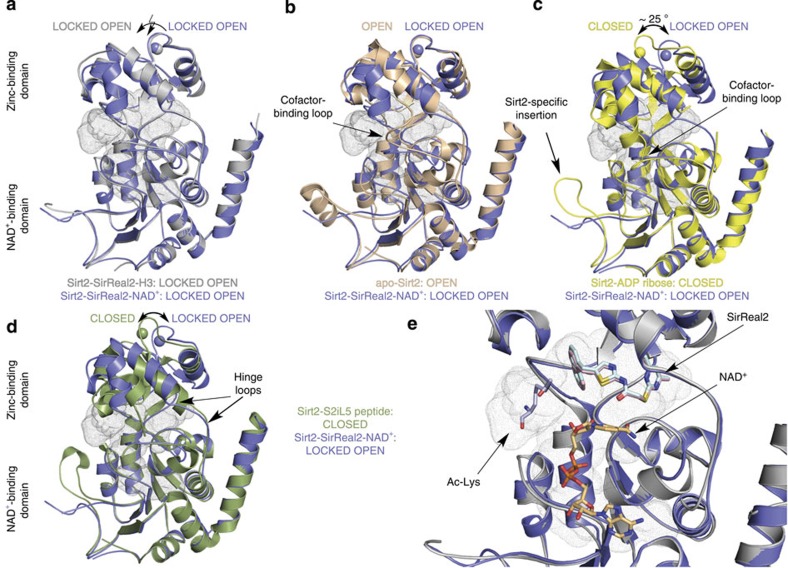
SirReal2 functions as a molecular wedge locking Sirt2 in an open conformation. (**a**) Overlay of Sirt2–SirReal2–NAD^+^ (slate blue) and Sirt2–SirReal2–H3 (light grey). Both structures are very similar (r.m.s.d. (C_α_ atoms)=0.8 Å) and feature an open conformation. The active site is indicated by small grey dots. (**b**) Superposition of Sirt2–SirReal2–NAD^+^ (slate blue) with Sirt2–apo (PDB-ID 3ZGO, salmon, residues 34–45 are omitted for better clarity). Both structures feature an open state despite major structural differences in the zinc-binding domain. (**c**) Superposition of Sirt2–SirReal2–NAD^+^ (slate blue) with the Sirt2-ADPR complex (PDB-ID 3ZGV, yellow, residues Tyr139-Gly141 of one hinge loop were not defined in the electron density map). The structures display major conformational differences in the orientation of the zinc-binding domain. While the ADPR complex is in a closed state, Sirt2–SirReal2–NAD^+^ adopts an open state. (**d**) Superposition of Sirt2–SirReal2–NAD^+^ with Sirt2 in complex with a macrocyclic peptide inhibitor S2iL5 (PDB-ID 4L3O, green). Similar to the Sirt2–ADPR complex, the Sirt2–S2iL5 complex assumes a closed conformation. While the Rossmann fold domain is very similar in both structures, major structural differences can be seen at the zinc-binding domain and at the Sirt2-specific insertion. (**e**) Close-up view on the active site using the superposition shown in **a**. SirReal2 (Sirt2–SirReal2–NAD^+^, light pink sticks; Sirt2–SirReal–H3, light cyan sticks) occupies the extended C-site of Sirt2. Binding of SirReal2 neither prevents binding of the acetyl-lysine substrate (light blue sticks) nor the cosubstrate NAD^+^ (light orange sticks). The cofactor-binding loop of both structures is omitted for clarity.

We solved the structure of Sirt2 in complex with SirReal2 and the cosubstrate NAD^+^ (structure termed Sirt2–SirReal2–NAD^+^) as well as in complex with SirReal2 and an acetyl-lysine peptide derived from histone H3 (residues 11–17, structure termed Sirt2–SirReal2-H3). Both Sirt2–SirReal2 crystals belonged to different monoclinic space groups and contained one monomer per asymmetric unit. They had the two-domain structure typical for sirtuins-a larger domain with a Rossmann fold and a smaller zinc-binding domain that are separated through a large groove that constitutes the active site (Fig. 2a). The structures are highly similar (root mean squared deviation, r.m.s.d. (C_α_ atoms)=0.8 Å) with the main differences in the cofactor-binding loop and its adjacent residues (r.m.s.d. (C_α_ residues 92–115)=1.3 Å). In addition, we observed the Sirt2-specific insertion (residues 289–304) that mediates crystal contacts as was reported for the Sirt2–ADPR complex (ADPR, PDB-ID 3ZGV^19^) and apo-Sirt2 (PDB-ID 1J8F, refined 3ZGO^18^,^19^). The cofactor-binding loop in both Sirt2–SirReal2 complexes adopts a conformation similar to the one observed in Sirt2 in complex with the product analogue ADPR (PDB-ID 3ZGV).

When compared with the available Sirt2 structures (apo-Sirt2: PDB-ID 1J8F, 3ZGO; Sirt2-ADPR: PDB-ID 3ZGV; Sirt2–S2iL5 peptide: PDB-ID 4L3O^28^) the zinc-binding domains in the Sirt2–SirReal2 structures adopt a conformation similar to the one in apo-Sirt2 (Fig. 2b–d, Supplementary Fig. 3a–c). On binding of the acetyl-lysine peptide substrate, the zinc-binding domain rotates towards the Rossmann fold domain. This has been termed as the ‘closure’ of the two domains and can be observed in several other human and bacterial sirtuin structures in complex with an acetyl-lysine peptide substrate^20^,^21^,^31^. This domain closure induces the formation of the acetyl-lysine-binding tunnel and the β-staple motif that mediates the acetyl-lysine peptide substrate-sirtuin interactions^32^. Despite the absence of an acetyl-lysine peptide substrate, the Sirt2–ADPR complexes also adopt the closed conformation. This is due to the Sirt2-specific insertion that acts as a pseudo-substrate in the crystal and binds to the acetyl-lysine-binding site of a neighbouring Sirt2 molecule.

Despite the ‘open’ conformation of the Sirt2–SirReal2 structures, SirReal2-inhibited Sirt2 adopts a substantially different structure from the one observed in Sirt2–apo (r.m.s.d. (C_α_ atoms)=1.4 Å, Fig. 2b), the complex of Sirt2 and ADPR (r.m.s.d. (C_α_ atoms)=1.6 Å, Fig. 2c) and the complex of Sirt2 and the S2iL5 peptide (r.m.s.d. (C_α_ atoms)=1.7 Å, Fig. 2d). Our structures feature a completely new and unexpected Sirt2 conformation, where SirReal2 functions as a ‘molecular wedge’ that traps Sirt2 in the open conformation even in the presence of an acetyl-lysine peptide substrate. We call this a ‘locked open’ state.

### SirReal2 occupies a yet-unexploited binding pocket

**Figure 3:**
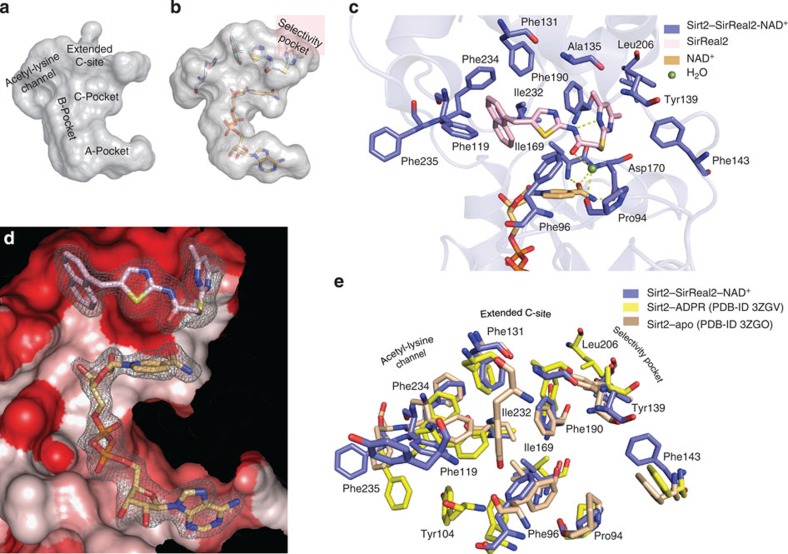
SirReal2 occupies the extended C-site and induces a major rearrangement of Sirt2’s active site. (**a**) Surface representation of the active site of apo-Sirt2, designating the individual subsites referred to in the text. (**b**) Orientation of SirReal2 in the active site of the Sirt2–SirReal2 complexes. The binding of SirReal2 induces the formation of the yet-unexploited selectivity pocket. (**c**) Interactions of SirReal2 (light pink) with Sirt2 in complex with the cosubstrate NAD^+^ (light orange). Interacting residues are represented as sticks (slate blue). Hydrogen bonds are shown as dashed yellow lines. (**d**) SirReal2 (light pink sticks, overall *B*-factor of 32.2 Å^2^) occupies the very hydrophobic extended C-site adjacent to the cosubstrate NAD^+^ (light orange sticks, overall *B*-factor of 41.8 Å^2^). The surface of Sirt2–SirReal2–NAD^+^ is coloured according to its hydrophobicity (red colour indicating increasing hydrophobicity). The σ-weighted 2*F*_o_−*F*_c_ electron density map is contoured at 1.0 *σ*. A stereo image of **d** is shown in Supplementary Fig. 4b. σ-weighted *F*_o_−*F*_c_ electron density OMIT maps for both ligands are shown in Supplementary Fig. 4d. (**e**) Comparison of the positions of the interacting residues of Sirt2–apo (PDB-ID 3ZGO, salmon), Sirt2–ADPR (PDB-ID 3ZGV, yellow) and Sirt2–SirReal2–NAD^+^ (slate blue). Residues are shown as sticks. The binding of SirReal2 results in a reorganization of the side chains of several residues. The most drastic side chain movement was observed for the residues that form the acetyl-lysine substrate channel and the selectivity pocket. The side chain of Tyr139 of Sirt2–ADPR complex was not defined in the electron density map. The position of the interacting residues of Sirt2 in complex with the macrocyclic peptide inhibitor S2iL5 are very similar to the ones of Sirt2–ADPR and are therefore not shown.

SirReal2 binds to the active site of Sirt2 (Figs 2e and 3) at the interface of the Rossmann fold domain and the zinc-binding domain, the site of the deacylation of ε-amino groups of lysines. The active site of Sirt2 has previously been divided into different sites (Fig. 3a). The A- and B-pocket, respectively, bind the ADPR moiety, whereas the C-pocket binds the NCA of NAD^+^. NAD^+^ is able to adopt different conformations. However, only a kinked conformation where the NCA moiety of NAD^+^ occupies the C-pocket is considered productive for catalytic deacylation. The hydrophobic acetyl-lysine-binding tunnel is formed by several phenylalanines and connects the NAD^+^-binding site to the acetyl-lysine-binding site. The pocket adjacent to the C-pocket has been termed extended C-site (EC-site)^33^.

The aminothiazole SirReal2 occupies this EC-site adjacent to the C-pocket, which is the physiological site for product inhibition by the feedback inhibitor NCA^30^ (Supplementary Fig. 2). It binds at this highly hydrophobic site in vicinity to the zinc-binding domain, where it does not interfere with the binding of NCA or the NCA moiety of NAD^+^ (Fig. 3b–d). The naphthyl moiety of SirReal2 protrudes into the substrate channel and the dimethylmercaptopyrimidine substituent (DMP) induces the formation of a binding pocket beyond the EC-site. This region is formed by two loops (residues 136–144, residues 188–191) of the hinge region that connect the Rossmann fold domain with the zinc-binding domain. We refer to this binding pocket in the following as the ‘selectivity pocket’. The position of SirReal2 in the EC-site of Sirt2 is very similar in both structures with either NAD^+^ or the acetyl-lysine peptide substrate (r.m.s.d. of 0.47 Å), and we will primarily describe the binding of SirReal2 in the presence of NAD^+^, as this structure likely represents the SirReal2-inhibited form of the enzyme. Structural comparison of the available Sirt2 structures with the Sirt2–SirReal2–H3 complex can be found in Supplementary Fig. 3.

Binding of SirReal2 to the EC-site is mainly driven by hydrophobic interactions (Fig. 3c,d). The naphthyl moiety of SirReal2 that protrudes into the acetyl-lysine-binding site is in van-der-Waals contacts with the NCA moiety of NAD^+^, Phe131, Leu134, Ile169, Ile232, Val233 and Phe234. In the selectivity pocket, the DMP moiety forms π–π-stacking interactions with Tyr139 and Phe190 in the selectivity pocket that is shaped by Ile93, Ala135, Leu138, Pro140, Phe143, Leu206 and Ile213. In addition, Pro94 hydrogen bonds via a structural water molecule (W40) to the carbonyl-O of SirReal2. Besides its interactions with the Sirt2 protein, the SirReal2 inhibitor also forms an internal hydrogen bond between the amide N–H and one of the pyrimidine nitrogens. This results in a rigid conformation with ideal complementarity to the active site of Sirt2.

### Binding of SirReal2 to Sirt2 rearranges the active site

The presence of SirReal2 results in a rearrangement of Sirt2’s active site. It is more pronounced in comparison with the Sirt2 structure in complex with ADPR (PDB-ID 3ZGV, Fig. 3e) than with the structure of Sirt2–apo (PDB-ID 3ZGO, Fig. 3e).

A site of major rearrangement is the selectivity pocket of the hinge region, where the DMP ring of SirReal2 is bound (Fig. 3e). Here the loop region from Lys136–Phe143 is substantially shifted upwards with respect to the Sirt2–apo structure and forms a lid above the DMP moiety. These residues seem to be more flexible, indicated by high *B*-factors, in structures of the closed conformation such as the Sirt2–ADPR or Sirt2–ADPR–NCA complexes (Supplementary Fig. 2) than in structures of the open conformation.

Another site of SirReal2-induced rearrangement is observed in the acetyl-lysine-binding site. Here the side chains of the residues forming the highly hydrophobic acetyl-lysine tunnel, Tyr104, Phe119, Phe131, Phe234 and Phe235 are shifted (Fig. 3e). In particular, the side chains of Phe235 and Tyr104 that usually cap the acetyl-lysine are rotated towards the surface of Sirt2. Surprisingly, this rearrangement does not prevent Sirt2 from binding its acetyl-lysine peptide substrate, but deacetylation and the domain closure is blocked effectively.

### Kinetic analyses of SirReal-mediated inhibition

**Figure 4:**
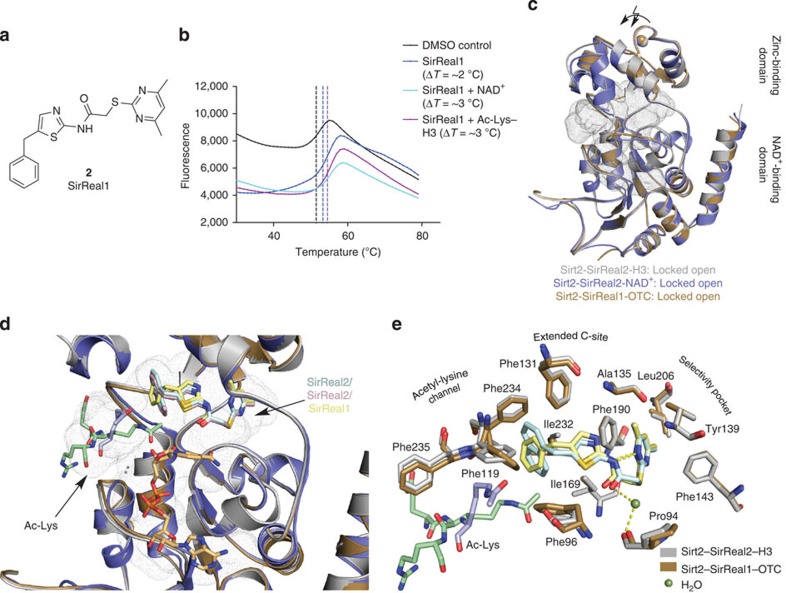
SirReal1 selectively inhibits Sirt2 and functions as a molecular wedge to lock Sirt2 in an open conformation. (**a**) Chemical structure of SirReal1 (**2**). (**b**) Representative thermal stability plots for Sirt2 in the presence of SirReal1 (50 μM) and either the cofactor NAD^+^ (5 mM) or an acetyl-lysine H3 peptide (5 mM). The presence of the cosubstrates enhances the stabilization of the Sirt2–SirReal1 complex (*n*=3). Representative thermal stability plots of Sirt2 in the absence of SirReal2 and the presence of NAD^+^ or an acetyl-lysine oligopeptide are shown in Supplementary Fig. 1d. (**c**) Overlay of Sirt2–SirReal1–OTC (brown) with Sirt2 structures in complex with SirReal2 (Sirt2–SirReal2–H3, light grey; Sirt2–SirReal2–NAD^+^, slate blue). All Sirt2–SirReal complexes share a high similarity (r.m.s.d. (C_α_ atoms)=0.44 Å to Sirt2–SirReal2–H3, 0.59 Å to Sirt2–SirReal2–NAD^+^) and represent the locked open conformation. The active site is represented as grey dots. (**d**,**e**) SirReal1 (light yellow sticks) occupies the extended C-site in a very similar fashion as observed for SirReal2 (light blue in Sirt2–SirReal2–H3, light pink in Sirt2–SirReal2–NAD^+^). Differences can be observed for the position of the side chains of Phe119, Phe235 and the acetyl-lysine peptides. The acetyl-lysine-binding site as well as the selectivity pocket are also the sites of major conformational changes compared with Sirt2–apo (PBD-ID 3ZGO) and Sirt2-ADPR (PDB-ID 3ZGV, see Fig. 3e). Hydrogen bonds are shown in dashed yellow lines. The cofactor-binding loop of **d** is omitted for clarity. A stereo image of the σ-weighted 2*F*_o_−*F*_c_ electron density maps for SirReal1 and the Ac-Lys-OTC oligopeptide as well as σ-weighted *F*_o_−*F*_c_ electron density OMIT maps of both ligands are shown in Supplementary Fig. 5b,d.

In the course of the investigation SirReal-mediated inhibition, we also determined the crystal structure of another aminothiazole, termed SirReal1 (**2**, Fig. 4a), in complex with Sirt2 and a different acetyl-lysine peptide substrate. The latter is derived from ornithine transcarbamoylase (OTC, structure termed Sirt2–SirReal1–OTC). SirReal1 has a benzyl instead of a naphthylmethyl substituent on the aminothiazole and is 26-fold less potent than SirReal2 in the same *in vitro* assay, but it retains high Sirt2 selectivity and shows similar behaviour in thermal stability assays (Supplementary Fig. 1b,c, Fig. 4b). Despite the presence of a different acetyl-lysine peptide, the structure of Sirt2–SirReal1–OTC bears a high resemblance to the Sirt2–SirReal2 complexes (r.m.s.d. (C_α_ atoms)=0.44 Å to Sirt2–SirReal2–H3, 0.59 Å to Sirt2–SirReal2–NAD^+^, Fig. 4c). SirReal1 also locks Sirt2 in the open conformation and shows an almost identical interaction pattern as observed for SirReal2 (Fig. 4d,e).

**Figure 5:**
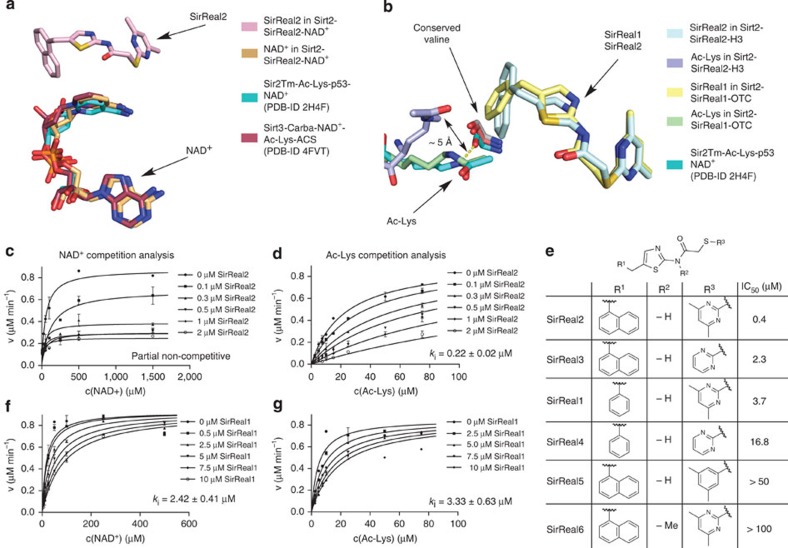
SirReal inhibitors suppress Sirt2 activity via a unique mechanism. (**a**) Superposition of Sirt2–SirReal2–NAD^+^ with ternary sirtuin complexes (Sir2Tm: PDB-ID 2H4F, aquamarine; Sirt3: PDB-ID 4FVT, raspberry) shows that NAD^+^ in Sirt2–SirReal2–NAD^+^ adopts a very similar kinked conformation to the one observed in ternary sirtuin complexes. The NCA ribose moiety of NAD^+^ in Sirt2–SirReal2–NAD^+^ shares more resemblance to the position of NCA ribose of NAD^+^ in 2H4F than to the one in 4FVT. (**b**) Overlay of Sirt2–SirReal2–H3, Sirt2–SirReal1–OTC with 2H4F of Fig. 4a. The bulky naphthyl moiety of SirReal2 forces the acetyl-lysine out of its physiological position, which can be seen in the ternary sirtuin complex of Sir2Tm, by ~5 Å. In this new position, the N_ε_-group of the acetyl-lysine of Sirt2–SirReal2–H3 cannot hydrogen bond to the backbone carbonyl-O of the conserved Val232. The less bulky benzyl moiety of SirReal1 allows a similar acetyl-lysine binding to Sirt2 as observed in the ternary complex of Sir2Tm. However, the presence of SirReal1 slightly enlarges the distance between the backbone carbonyl-O of Val233 to the N_ε_ of the acetyl-lysine, thus disabling hydrogen bond formation. (**c**,**d**,**f**,**g**) Competition analyses of SirReal1/2-mediated inhibition. SirReal2 is a partial non-competitive inhibitor towards NAD^+^, while SirReal1 functions as a NAD^+^-competitive inhibitor. SirReal1 and SirReal2 are both competitive to the acetyl-lysine peptide. Data are presented as mean±s.d. (*n*=2). (**e**) Structure–activity relationships (SAR) for SirReal inhibitors. Only the combination of the bulky naphthyl moiety with the DMP and the non-methylated amide results in submicromolar Sirt2 inhibition. Inhibition data were determined using the substrate ZMAL (*n*=3).

To get insights into the inhibition mechanism, we first compared the structures of Sirt2–SirReal complexes with the available sirtuin structures lacking inhibitors. For the cosubstrate NAD^+^, the binding mode does not differ substantially. NAD^+^ of Sirt2–SirReal2–NAD^+^ also adopts a kinked conformation with a similar network of hydrophilic and hydrophobic interactions as observed for NAD^+^ or Carba-NAD^+^ in ternary sirtuin complexes (PDB-ID 2H4F^34^, Sir2Tm-Ac-Lys-p53-peptide-NAD^+^, PDB-ID 4FVT^23^, Sirt3-Carba-NAD^+^-Ac-Lys-ACS-peptide, Fig. 5a). As the main difference, the NCA ribose moiety of Carba-NAD^+^ is rotated ~30° around its glycosidic bond compared with NAD^+^ in Sirt2–SirReal2–NAD^+^ or Sir2Tm-Ac-Lys-p53-peptide-NAD^+^. The binding mode of NAD^+^ in Sirt2–SirReal2–NAD^+^ therefore shares a higher resemblance to the conformation of NAD^+^ in the ternary complex with Sir2Tm.

The acetyl-lysine-binding modes in ternary sirtuin complexes and Sirt2–SirReal2 structures on the other hand show substantial differences (Fig. 5b). In uninhibited sirtuin-acetyl-lysine-peptide structures, the acetyl-lysine-containing peptide binds in the cleft between the zinc-binding and NAD^+^-binding domain, respectively, inserting its acetyl-lysine into a hydrophobic tunnel that is formed by several highly conserved phenylalanines. The binding of the acetyl-lysine is further stabilized by a hydrogen bond between the N_ε_–H of the acetyl-lysine and the backbone carbonyl-O of a conserved valine. The hydrophobic acetyl-lysine–binding tunnel is not formed in all Sirt2–SirReal structures, since Phe235, which usually caps the acetyl-lysine, is rotated ~90° towards the surface. Moreover, the bulky naphthyl moiety of SirReal2 forces the acetyl-lysine ~5 Å out of its physiological position, which can be seen in ternary complexes of sirtuins (Sir2Tm-Ac-Lys-p53-peptide-NAD^+^, PDB-ID 2H4F, Sirt3-Ac-Lys-ACS-peptide-Carba-NAD^+^, PDB-ID 4FVT, Fig. 5b).

In the Sirt2 complex with SirReal1, the acetyl-lysine-binding mode is different. The acetyl-lysine adopts an almost physiological position, as it is observed in the above-mentioned ternary sirtuin complexes. However, even in case of SirReal1, the benzyl moiety of SirReal1 shifts the acetyl-lysine of the OTC peptide towards His187, weakening the formation of the hydrogen bond between the N_ε_–H of the acetyl-lysine and the backbone carbonyl-O of the conserved valine (distance between carbonyl-O of valine and N_ε_-KAc: 3.2 Å in Sirt2–SirReal1–OTC, 2.5–2.7 Å in ternary complexes).

To investigate the SirReal-mediated inhibition kinetics, we performed competition analyses for SirReal1 and SirReal2 (Fig. 5c,d,f and g). SirReal2 is partially non-competitive and SirReal1 is competitive towards NAD^+^ (Fig. 5c,f). Both SirReal inhibitors also exhibit acetyl-lysine competition with inhibition constants of 0.22 μM for SirReal2 and 3.33 μM for SirReal1 (Fig. 5d,g). This is in line with the protrusion into the acetyl-lysine-binding site that was seen in the crystal structures. However, despite competition towards acetyl-lysine substrates or the cosubstrate NAD^+^, the presence of SirReal1/2 inhibitors does not disable Sirt2 to bind its substrates.

For further exploration of the SirReal-mediated Sirt2 inhibition, we synthesized several SirReal derivatives (SirReal3–6, Supplementary Fig. 5, Supplementary Notes) and determined their inhibitory potencies (Fig. 5e). Only the combined presence of the naphthyl substituent with the DMP moiety and the non-methylated amide leads to a submicromolar Sirt2 inhibition. The substitution of the DMP moiety with a dimethylmercaptophenyl substituent or the methylation of the amide-nitrogen results in a significant loss of inhibition (>100-fold). This suggests that the formation of the intramolecular hydrogen bond and the resulting structural rigidity of Sirt2-bound SirReal2 are indispensable for potent Sirt2 inhibition.

### Structural aspects of isotype-selective inhibition

One striking feature of the SirReal2-mediated inhibition is its isotype selectivity. SirReal2 inhibits Sirt2 >1,000-fold more potently than Sirt1, Sirt3, Sirt4, Sirt5 and Sirt6 and it is therefore one of the most selective sirtuin inhibitors known to date.

**Figure 6:**
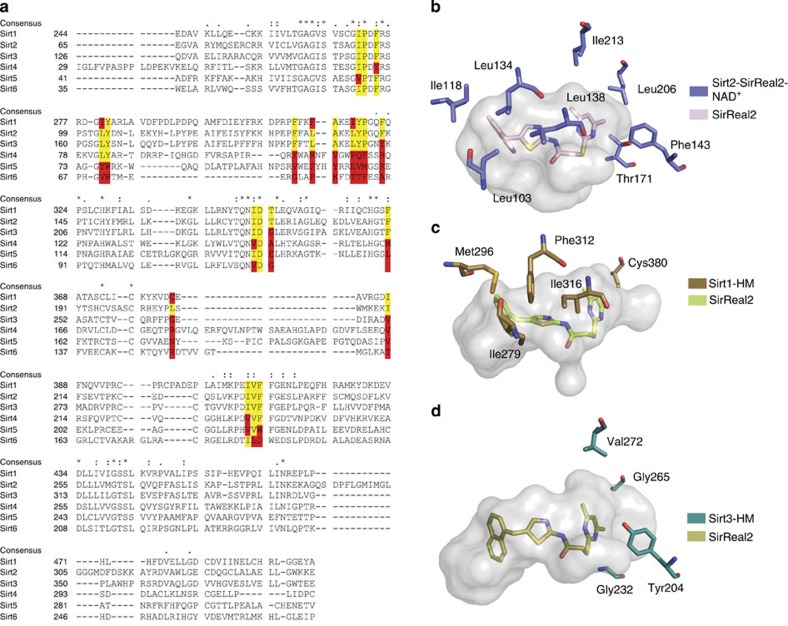
SirReal2 selectively inhibits Sirt2 via a Sirt2-specific amino acid network. (**a**) Structural sequence alignment of the Sirt1–6 deacylase domain. The residues that presumably interact with SirReal2 are highlighted in yellow if they are equivalent to the residues of Sirt2. If they differ from the residues of Sirt2, they are highlighted in red. The structural sequence alignment was generated using T-Coffee^35^ and slightly modified. (**b**–**d**) Surface representation of the binding pockets of SirReal2 in Sirt2–SirReal2–NAD^+^ and in the homology models of Sirt1 (Sirt1-HM) and Sirt3 (Sirt3-HM). The residues that differ in the three isotypes are represented as sticks (Sirt2, slate blue; Sirt1, brown; Sirt3, turquoise). SirReal2 is shown as light pink sticks (Sirt2–SirReal2–NAD^+^), lime sticks (Sirt1-HM) and olive sticks (Sirt3-HM). Despite the highly conserved active site, the binding pockets appear in very different shapes due to differences in the amino acid sequence. In case of Sirt1 the amino acids that contribute to an unfavourable binding of SirReal2 are Ile279, Met296, Phe312, Ile316 and Cys380. In the case of Sirt3, the amino acids are Tyr204, Gly232, Gly265 and Val272.

To analyse the basis of this high isotype selectivity, we created a structural sequence alignment of the deacylase domain of Sirt1–6 and compared the crystal structure of the Sirt2–SirReal2 complex with available crystal structures of sirtuins in their open conformation (Fig. 6a, Supplementary Fig. 7a,b)^35^. Assuming that SirReal2 binds to the other sirtuin isotypes in a similar fashion as observed for Sirt2, Sirt4–6 exhibit major differences in their amino acid sequence. The structural differences are also very pronounced (Supplementary Fig. 7a) rationalizing the observed lacking *in vitro* inhibition of Sirt4–6 by SirReal2. Sirt1 and Sirt3, on the other hand, are phylogenetically more closely related to Sirt2 and show only minor sequence variations^36^. Their conformation is more similar to the Sirt2–SirReal2–NAD^+^ complex than to the conformation of the isotypes Sirt5/6 (Supplementary Fig. 7b). But they still show major structural differences (r.m.s.d. (C_α_ atoms)=1.6 Å). As it was not possible to dock SirReal2 in any of the available Sirt1 and Sirt3 X-ray crystal structures (Supplementary Methods), we wanted to probe whether Sirt1 and Sirt3 were able to adopt a similar conformation as observed in the Sirt2–SirReal2 structures that would allow binding of SirReal2. This would enable us to see whether the minor sequence variations within the deacylase domain of Sirt1–3 would have an influence on SirReal2 binding. Therefore, we generated homology models of Sirt1 (Sirt1-HM) and Sirt3 (Sirt3-HM) based on our Sirt2–SirReal2 structures (Supplementary Methods). Stereochemical analyses as well as molecular dynamics simulations indicated high-quality model structures, and it was indeed possible to dock SirReal2 into these homology models (Supplementary Fig. 7c–h). However, the docking poses of SirReal2 in Sirt1-HM and Sirt3-HM gave less favourable docking scores compared with the requisite scores for the docking poses of SirReal2 in Sirt2–SirReal2 structures. Here the position and the conformation of SirReal2 were correctly predicted (Fig. 6b). In case of Sirt1, residues Leu103, Ile118, Leu134, Leu138 and Leu206 of Sirt2 are substituted with Ile279, Met296, Phe312, Ile316 and Cys380 (Fig. 6c). Cys380 gives the hypothetical selectivity pocket of SirReal2 in Sirt1-HM a very different shape and changes its surface characteristics. The bulky Phe312 and Ile316 as well as Met296 and Ile279 also tighten the EC-site, resulting in an unfavourable orientation of the aminothiazole and naphthyl moieties in possible docking poses. In the case of Sirt3, the differences are mainly located at the selectivity pocket. Here Phe143, Thr171, Leu206 and Ile213 of Sirt2 are substituted by Tyr204, Gly232, Gly265 and Val272 (Fig. 6d). The less bulky Gly232, Gly265, Val272 of Sirt3 form a much wider and also more solvent-accessible selectivity pocket as compared with the Sirt2–SirReal2 structures. In contrast to the SirReal2-binding pockets of the homology models of Sirt1 and Sirt3, SirReal2 bound to Sirt2 can adopt a conformation that is in almost perfect complementarity with the protein, which is stabilized by the intramolecular hydrogen bond between the DMP substituent and the amide. This is not possible in Sirt1 and Sirt3 and also rationalizes the observed isotype selectivity.

### *In vivo* inhibition of Sirt2

**Figure 7:**
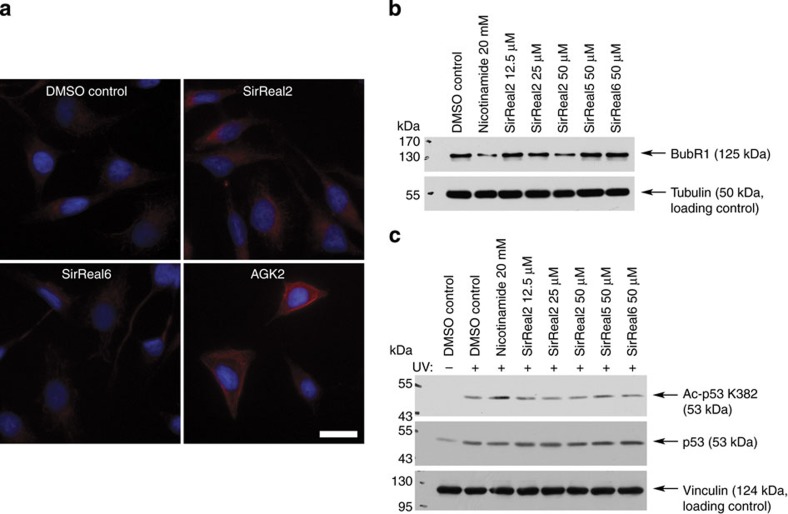
SirReal2 inhibits Sirt2 *in vivo*. (**a**) Acetylation level of the microtubule network (red) in the presence or absence of SirReal2 at a concentration of 20 μM. Treatment with SirReal2 leads to hyperacetylation of the microtubule network in a similar manner as observed for the Sirt2 inhibitor AGK2. Treatment with SirReal6 on the other hand results in no substantial change of acetylation level. The scale bar represents 5 μm (*n*=4). (**b**) Abundance of BubR1 after incubation with SirReal2 (*n*=3). (**c**) SirReal2 does not alter p53-Lys382-acetylation after ultraviolet damage (*n*=3). Raw immunofluorescent images and uncropped blots are shown in the Supplementary Figs 8–10.

To validate that SirReal2 could be used as a tool to investigate the effects of Sirt2 inhibition in a cellular setting, we incubated HeLa cells with SirReal2 at various concentrations and determined the level of α-tubulin acetylation (Supplementary Fig. 8a,b). Incubation with SirReal2, but not with SirReal6, resulted in a significant increase of α-tubulin acetylation consistent with an *in vivo* inhibition of Sirt2 as shown previously^37^. These changes are not as pronounced when compared with the changes induced by the inhibition of the other main tubulin deacetylase KDAC6 (refs 38, 39). KDAC6 activity is not affected *in vitro* in the presence of SirReal2 (Supplementary Fig. 8c). To verify the observations from the western blot data, we also visualized the acetylation level by means of immunofluorescence microscopy. Again, the incubation with SirReal2 resulted in a partial increase of the acetylation of the microtubule network similar to the effects observed after treatment with the Sirt2-inhibitor AGK2 (Fig. 7a, Supplementary Fig. 9)^39^,^40^. SirReal6, on the other hand, does not alter microtubule acetylation. In addition, we analysed another Sirt2 target. Recently, we reported that the stability of spindle assembly checkpoint protein BubR1 is under control of Sirt2 (ref. 41). A decline in BubR1 over time has been linked to mammalian aging^42^. Deacetylation of Lys668 of BubR1 by Sirt2 inhibits the ubiquitination of BubR1 and its designation to the proteasome. Therefore, the abundance of BubR1 can be used as a functional measure for *in vivo* Sirt2 inhibition. Incubation with SirReal2 indeed significantly resulted in a dose-dependent depletion of BubR1, whereas SirReal5/6 did not influence BubR1 concentrations (Fig. 7b, Supplementary Fig. 8d,e). We also found that SirReal2 treatment did not alter cell cycle distribution, ruling out that the effect on BubR1 was indirect through induction of cell cycle changes (Supplementary Fig. 8f). To determine if SirReal2 selectively inhibits Sirt2 *in vivo*, we assessed p53 acetylation following genotoxic stress (Fig. 7c, Supplementary Fig. 10a). Acetylation of p53 occurs in response to ultraviolet exposure to cells^43^ and this acetylation is regulated, in part, by the isotype Sirt1 (refs 44, 45). On exposure to ultraviolet light, we detected an increase in acetylation of p53, which was further increased on treatment with the pan-sirtuin inhibitor NCA. However, we did not observe an increase in acetylation of p53 following treatment with SirReal2/5/6. Similarly, we also tested if SirReal2 could inhibit Sirt3 in cells by assessing mitochondrial protein acetylation. Sirt3 has previously been demonstrated to regulate global mitochondrial protein acetylation^46^. Following treatment with SirReal2 or NCA as a positive control, we purified mitochondria and assessed protein acetylation by western blotting. We found that treatment with NCA leads to an increase in the acetylation of mitochondrial proteins, whereas treatment with SirReal2 did not, suggesting that SirReal2 is unable to regulate Sirt3 activity in cells (Supplementary Fig. 10b). These results confirm the *in vitro* observations and indicate that SirReal2 has a strong specificity towards Sirt2 *in vivo* when compared with the other members of Class-I sirtuins Sirt1 and Sirt3.

## Discussion

There are many indications that sirtuins play an important role in neurodegeneration, cancer, bacterial infections and inflammation and that a modulation of Sirt2 activity could be a new strategy for pharmaceutical intervention. However, the physiological functions of Sirt2 are far from being completely understood and conclusive evidence for the suitability of Sirt2 as a pharmaceutical target is, at least in some cases, missing. To further explore Sirt2 function in a cellular environment, there is a definite need for selective and potent Sirt2 modulators.

So far, most sirtuin modulators lack either potency, selectivity or drug-like physicochemical properties. Recent screening campaigns have identified several potent and/or selective inhibitors^15^,^24^,^25^,^26^; however, with the exception of the macrocyclic peptide S2iL5, it is not clear how these inhibitors bind to Sirt2. And although several X-ray structures of sirtuins in complex with inhibitors have been reported lately^28^,^33^,^47^,^48^,^49^,^50^,^51^, these structures do little to reveal a rationale for a Sirt2-selective inhibition.

With the identification of the SirReal inhibitors, we establish the structural basis for Sirt2-selective inhibition and report a new potent Sirt2-selective inhibitor scaffold. As noted above, the intramolecular hydrogen bond between the amide of the aminothiazole and a nitrogen atom of the DMP moiety gives the inhibitor a rigid form that can act as a molecular wedge locking the enzyme conformation with subsequent Sirt2 inhibition. Not only essential for the potency, the intramolecular bond also seems to play an important role for the compound’s Sirt2 selectivity, as it can only be formed when bound in perfect complementarity to the active site of Sirt2. This seems not to be possible if bound to Sirt1 or Sirt3.

Another important aspect alongside the internal hydrogen bond of SirReal-mediated Sirt2 inhibition is the exploitation of the selectivity pocket by SirReal inhibitors. The only other isotype-selective sirtuin inhibitors with known structures, CHIC-35 (Sirt1-selective)^48^, EX-527 (Sirt1-selective)^33^ and SRT1720 (Sirt3-selective)^49^ either bind to the EC-site and/or to the acetyl-lysine-binding site but neither the indole inhibitors nor SRT1720 or any of the inhibitors whose binding modes have been elucidated by means of X-ray crystallography occupy the selectivity pocket (Fig. 6). This pocket is formed by two loops that connect the Rossmann fold domain with the smaller zinc-binding domain. The residues that form this pocket significantly differ within the sirtuin family and targeting this pocket may present a new strategy for selective sirtuin inhibitor design. These particular findings would not have been discovered with the use of computational methods and the available Sirt2 structures.

In conclusion, with the identification of SirReal2, we provide an isotype-selective drug-like inhibitor with optimized potency and physicochemical properties in comparison with previously published Sirt2 inhibitors (Supplementary Table 1). We established valuable structural insights into selective Sirt2 inhibition and show that SirReal2 inhibits Sirt2 *in vivo* without affecting the activity of the other Class-I sirtuins Sirt1 and Sirt3. The observed selectivity towards Sirt3 may, in part, stem from a lack of penetration into the mitochondria but the cellular net result is as desired. SirReal2 may therefore be used for further cellular studies to probe Sirt2 biology. Our findings may constitute the basis for further selective sirtuin inhibitor development and provide a new tool for sirtuin biology.

## Methods

### Cloning

The gene sequences coding for human Sirt2_56–356_ (Uniprot: Q8IXJ6) or human Sirt3_118–395_ (Uniprot: Q9NTG) were cloned in a modified pET15b vector that contained His_10_-Tag instead of a His_6_-Tag and a cleavage site for TEV protease instead of one for thrombin.

### Protein expression and purification

Human Sirt1_133–747_ (Uniprot: Q96EB6), human Sirt2_25–389_, human Sirt3_101–399_, human Sirt5_34–302_ (Uniprot: Q9NXA8) and human Sirt6_13–308_ (Uniprot: Q8N6T7) were purified as described before^47^,^52^,^53^. Human Sirt4_25–314_ (Uniprot: Q9Y6E7) was expressed and purified as described before^54^ with the exception that autoinduction with 0.2% (w/v) lactose in TB media was used for expression.

Sirt2_56–356_ and Sirt3_118–395_ were expressed in *E. coli* strain BL21(DE3)Codonplus RIPL cells overnight at 18 °C. Overexpression was induced with isopropyl-β-D-thiogalactoside (0.1 mM) at an OD_600_ of 0.6. Cells were harvested, resuspended in lysis buffer (Sirt2_56–356_: 50 mM Tris/HCl, 500 mM NaCl, 5% (v/v) glycerol, 5 mM β-mercaptoethanol, pH 8.0; Sirt3_118–395_: 50 mM HEPES, 500 mM NaCl, 5% (v/v) glycerol, 5 mM β-mercaptoethanol, pH 7.5) and lysed using a microfluidizer (Microfluidics). After the removal of cell debris, the supernatant was applied to a HisTrapFF 5 ml column (GE Healthcare), washed intensively before TEV protease (excess) was applied directly on the column. After an overnight digestion at 4 °C, the digested protein was eluted with lysis buffer, concentrated and further purified using a Superdex S75 26/60 gel filtration column (Sirt2_56–356_: 25 mM Tris/HCl, 150 mM NaCl, pH 8.0; Sirt3_118–395_: 25 mM HEPES, 200 mM NaCl, 5% (v/v) glycerol, 5 mM β-mercaptoethanol, pH 7.5). Sirtuin-containing fractions were collected and concentrated to 20 mg ml^−1^. All purification steps were monitored using SDS-polyacrylamide gel electrophoresis (SDS–PAGE). Protein concentration was determined by the Bradford assay (Roth).

### *In vitro* sirtuin assay

Initial screens were conducted with a high-throughput fluorescence-based assay using the substrate ZMAL (Z-Lys(Acetyl)-AMC) that was synthesized according to published procedures^29^. For this human Sirt1_133–747_, human Sirt2_25–389_, human Sirt3_101–399_ or Sirt3_118–395_ were mixed with assay buffer (50 mM Tris/HCl, 137 mM NaCl, 2.7 mM KCl, 1 mM MgCl_2_, pH 8.0), β-NAD^+^ (final assay concentration 500 μM), the substrate ZMAL (final assay concentration 10.5 μM from a 12.6 mM stock solution in DMSO) and the respective inhibitor in DMSO at various concentrations or DMSO as a control (final DMSO concentration 5–20% (v/v)). The mixture was incubated at 37 °C for 4 h, with agitation at 150 r.p.m. Deacetylation was then stopped by the addition of a solution containing NCA and trypsin (50 mM Tris/HCl, 100 mM NaCl, 6.7% (v/v) DMSO, trypsin 5.5 U μl^−1^, 8 mM NCA, pH 8.0, 60 μl) and the mixture was then incubated for tryptic digestion of the deacetylated product to release the fluorophor (20 min, 37 °C, 150 r.p.m.). Then the fluorescence intensity was measured in a microplate reader (BMG Polarstar, *λ*_ex_ 390 nm, *λ*_em_ 460 nm). The amount of inhibition was determined with respect to the mixture with only DMSO. IC_50_ values were determined with Graphpad Prism software using a non-linear regression to fit the dose–response curve. SirReal1 and SirReal2 were also tested for Sirt1–3 inhibition with a non-labelled acetyl-lysine peptide substrate (based on α-tubulin with two additional tryptophans (residues 36–44, H-PSDK(Acetyl)TIGGWW-NH_2,_ 10 μM, Supplementary Methods). SirReal2 was also tested for Sirt5–6 inhibition with non-labelled acyl-lysine peptide substrates (Sirt5: Benzoyl-GVLK(Succinyl)EYGV-NH_2_, 10 μM; Sirt6: Ac-EALPKK(Myristoyl)TGG-NH_2_, 10 μM) The substrate was incubated (10 min, ~0.5 μM Sirt1/2/3/5/6, 500 μM β-NAD^+^, 5–20% (v/v) DMSO, 50 mM Tris/HCl, 137 mM NaCl, 2.7 mM KCl, 1 mM MgCl_2_, pH 8.0), stopped by the addition of trifluoroacetic acid (TFA, 10% (v/v), final concentration 1% (v/v)). The components of the stopped reaction mixture were separated by HPLC (Agilent 1100, Phenomenex reversed phase column Kinetex RP18 2.7 μm, 50 × 3 mm) using a linear gradient of acetonitrile (20–60% (v/v) acetonitrile, 0.1% (v/v) TFA, 0.6 ml min^−1^). Peaks of acetylated and deacetylated substrate were quantified by absorption at 280 nm. Sirt4-dependent deacetylation reactions were performed with an acetylated Nnt397-peptide (H-NITKLLK(Acetyl)AISPDK-NH_2_, 250 μM, GL Biochem., in 50 mM Tris/HCl, 150 mM NaCl, pH 7.5). Samples were taken between 0 and 45 min and reactions were stopped by mixing 1:1 with 0.5% (v/v) TFA. The samples were then diluted to a peptide concentration of 5 μM with 0.1% (v/v) formic acid and analysed on an EASY-nLCII connected to a LTQ mass spectrometer (Thermo Fisher Scientific). Peptides were separated by a linear gradient of acetonitrile (0–100% (v/v), 0.1% (v/v) TFA, 300 nl min^−1^) on a reprosil C18 reversed phase column. Peak areas of acetylated and deacetylated peptides were extracted using Skyline^55^. A solution with DMSO was used as a negative control while a solution with the physiological inhibitor NCA served as a positive control (Fig. 1c). Owing to the lack of a suitable screening assay for human Sirt7, we focused our studies on isotypes Sirt1–6. Fluor-de-Lys assays (Enzo Life Sciences) were conducted according to the manual using the HDAC8 Fluor-de-Lys deacetylase substrate (BML-KI178-0005).

### Protein crystallization

All crystallization trials were performed in 96-well plates (Intelli-Plate 96-3 Low Profile, Art Robbins Instruments) using an Oryx nano pipetting robot (Douglas Instruments). Reservoir solutions were precooled to 4 °C and screens were then pipetted at 20 °C.

For co-crystallization experiments with SirReal1 and SirReal2, a solution of the truncated Sirt2_56–356_ (20 mg ml^−1^ final concentration) was preincubated with a saturated aminothiazole solution (100 mM stock solution in DMSO, 1–2% (v/v) DMSO final concentration) in the presence of H3 peptide (100 mM stock solution in 25 mM HEPES, 200 mM NaCl, 5% (v/v) glycerol, pH 7.5, 2 mM final concentration, derived from acetylated Histone H3 (residues 11–17), Peptide Specialty Laboratories), acetylated OTC oligopeptide (100 mM stock solution in DMSO, 2 mM final concentration, derived from ornithine transcarbamoylase (residues 83–92), Peptide Specialty Laboratories) or β-NAD^+^ (Sigma-Aldrich, 100 mM stock solution in 25 mM Tris/HCl, 150 mM NaCl, pH 8.0, 10 mM final concentration) for 60 min at 4 °C, centrifuged (4 °C, 10 min, 16,100 *g*) and crystallized. Sirt2–SirReal1–OTC–peptide complex crystallized in 25% (w/v) polyethylene glycol (PEG) 3,350 and 0.1 M Bis–Tris buffer at pH of 6.5 and 4 °C, Sirt2–SirReal2–H3–peptide complex crystallized in 2.8 M (NH_4_)_2_SO_4_ and 0.1 M Tris/HCl buffer at pH 9.0 and 4 °C. For both conditions, a protein solution to reservoir solution ratio of 1:3 was used. The Sirt2–SirReal2–NAD^+^ complex crystallized in 0.1 M KSCN, 30% (w/v) PEG MME 2,000 at 4 °C with a protein solution to reservoir solution ratio of 3:1. The crystals were mounted in nylon loops and if required, cryoprotected by the addition of 20% (v/v) of glycerol before flash cooling in liquid nitrogen.

Crystals of Sirt2 in complex with ADPR (13 mg ml^−1^ final concentration, 20 mM ADPR, 1 M stock solution in 1 M Tris/HCl buffer at pH 9.0) were obtained using 18% (w/v) PEG 10,000 and 0.1 M Bis–Tris buffer at pH 5.75 and 20 °C. The crystals formed after 1 day and were then soaked in a buffer containing 18% (w/v) PEG 10,000, 0.1 M Bis–Tris buffer at pH 5.75, 200 mM NCA for 30 min. They were then mounted in nylon loops after addition of glycerol to a final concentration of 20% (v/v) before flash cooling in liquid nitrogen.

### Data collection and processing

Data were collected at beamline X06SA (Sirt2–SirReal2–H3 complex) or X06DA (Sirt2–SirReal1–OTC complex, Sirt2–ADPR–NCA complex) with Pilatus detectors (Dectris) at the Swiss Light Source (Villigen, Switzerland). All experiments were carried out at 100 K with oscillations of 0.25 or 0.5° at an X-ray wavelength of 1.0 Å. The Sirt2–SirReal2–NAD^+^ complex was determined from data collected on a MicroMax 007HF rotating anode X-ray generator (Rigaku) at an X-ray wavelength of 1.5418 Å, equipped with a mar345 image plate detector (Mar Research). All data sets were processed with Imosflm^56^ or XDS^57^ and scaled based on the CC* criterion^58^ using Aimless^56^.

### Structure solution and refinement

All structures were solved by molecular replacement using either MOLREP^59^ or PHASER^60^ with a monomer of Sirt2–ADPR (PDB-ID 3ZGV)^19^ or the initial Sirt2-aminothiazole-structure (Sirt2–SirReal2–H3) as a search model. Model building was carried out using Coot^61^ and the structure was refined with REFMAC5 (ref. 62). Ligands were generated using the Grade Web Server (Global Phasing Ltd., Cambridge). All structures were validated using the Molprobity server^63^.

Sirt2–SirReal1–OTC was refined to a final *R*_work_ of 26.0% and *R*_free_ of 28.2% with 98% of all amino acids of the refined model found in the most favored regions of the Ramachandran plot. Sirt2–SirReal2–H3 was refined to a final *R*_work_ of 18.1% and *R*_free_ of 18.8% with 98% of all residues falling into the most-favoured region of the Ramachandran plot. The Sirt2–SirReal2–NAD^+^ complex was refined to a final *R*_work_ of 20.2% and *R*_free_ of 24.7% with 97% of all residues in the most-favoured Ramachandran plot regions. Finally, the Sirt2–ADPR–NCA complex was refined to a final *R*_work_ of 21.1% and *R*_free_ of 23.9% with 98% of all residues in the most favored Ramachandran plot regions. All structures do not have Ramachandran outliers. Further data collection and refinement statistics are found in Table 1 (Sirt2–SirReal1–OTC, Sirt2–SirReal2–H3, Sirt2–SirReal2–NAD^+^) and in Supplementary Table 2 (Sirt2–ADPR–NCA).

Most of the residues of all new structures are well defined in the electron density maps except for some parts of the flexible cofactor-binding loop (residues 96–120, Sirt2–SirReal2–NAD^+^, Sirt2–SirReal2–H3, Sirt2–SirReal1–OTC) and parts of the Sirt2-specific insertion loop (residues 295–305, Sirt2–SirReal2–NAD^+^, Sirt2–SirReal2–H3, Sirt2–SirReal1–OTC). The weak density for NAD^+^ in the Sirt2–SirReal2–NAD^+^ complex may also be due to partial hydrolysis of NAD^+^ and the formation of different conformers. The visible amino-terminal histidine and methionine visible in the Sirt2–ADPR–NCA complex and Sirt2–SirReal2–NAD^+^ complex structures originate from the *NdeI* restriction site of the modified pET15b vector. σ-weighted *F*_o_−*F*_c_ electron density OMIT maps were generated with the Phenix suite^64^. Images were prepared with Pymol (The Pymol Molecular Graphics System, Version 1.6, Schrödinger, LLC), r.m.s.d. values were calculated with SUPERPOSE^65^ and the surface of the binding pockets was generated using HOLLOW^66^. The two-dimensional representation of the interactions of NCA with Sirt2 were generated with LigPlot+^67^.

### Determination of kinetic parameters for SirReal1/2

For SirReal-inhibition kinetics, a mixture of α-tubulin peptide substrate (residues 36–44, H-PSDK(Acetyl)TIGGWW-NH_2,_ 2.5–75 μM, for details about the α-tubulin peptide synthesis see Supplementary Methods), β-NAD^+^ (5–1,500 μM), SirReal1/2 (various concentrations in 50 mM Tris/HCl, 137 mM NaCl, 2.7 mM KCl, 1 mM MgCl_2_, 5% (v/v) DMSO, pH 8.0) was incubated (37 °C). The reaction was started by the addition of human Sirt2_56–356_ (2 μM), stopped after 1–50 min with TFA (10% (v/v), final concentration 1% (v/v)) and analysed by HPLC as described above. The peak areas were integrated and converted to initial velocities calculated from the peak areas as the fraction of deacetylated peptide from total peptide. From this, reaction rates in μM min^−1^ were obtained by linear regression, while *K*_m_ and *k*_cat_ were obtained directly from Michaelis–Menten plots using Graphpad Prism software. β-NAD^+^ (500 μM) was used for the determination of the kinetic parameters for the peptide substrate, 100 μM of peptide substrate was used for the determination of the kinetic parameters for β-NAD^+^.

### Thermal shift assays

Human Sirt2_56–356_ (0.2 mg ml^−1^ final concentration) was mixed with or without ligand containing buffer (25 mM Tris/HCl, 150 mM NaCl, 5% (v/v) DMSO, 1:4,000 Sypro Orange, pH 8.0) in absence or presence of β-NAD^+^ (100 mM stock solution in 25 mM Tris/HCl, 150 mM NaCl, pH 8.0, final assay concentration 5 mM) or acetylated H3-peptide (100 mM stock solution in 25 mM HEPES, 200 mM NaCl, 5% (v/v) glycerol, pH 7.5, final assay concentration 5 mM). Fluorescence was monitored during a temperature ramp from 25–95 °C (1 °C min^−1^) using a Bio-Rad iCycler iQ5 (4titude, FrameStar 96-well plates, 4ti-0771, 4titude qPCR Seal, 4ti-0560). Melting temperatures were determined according to published procedures^68^ using Graphpad Prism software.

### Cell cultivation

HeLa cells (DSMZ accession no. 057) and U2OS cells (ATCC accession no. HTB-96) were grown in Dulbecco’s modified Eagle’s medium (PAA) containing 10% (v/v) fetal calf serum (FCS, PAA), 1% (v/v) penicillin (PAA), 1% (v/v) streptomycin (PAA), 1% (v/v), L-glutamine (PAA) at 37 °C in a 5% (v/v) CO_2_ atmosphere.

### Tubulin acetylation

HeLa cells were plated in petri dishes (5 cm, PAA), incubated overnight to a confluency of 30–40% and then treated with SirReal2 dissolved in RPMI1640 medium supplemented with fresh 20% (v/v) FCS (PAA), 1% (v/v) penicillin (PAA), 1% (v/v) streptomycin (PAA), 1% (v/v), L-glutamine (PAA), 1% (v/v) DMSO for 5 h at various concentrations. Cells were then washed with prewarmed PBS (2 ml), lysed in SDS–PAGE sample buffer (70 μl, 50 mM Tris/HCl, 0.5 mM EDTA, 1 × Complete Protease Inhibitors (Sigma-Aldrich), 2% (v/v) IGEPAL (Sigma-Aldrich), 2% (w/v) SDS, 10% (v/v) glycerol, 50 mM NCA (Sigma-Aldrich), 3.3 μM trichostatin A (Sigma-Aldrich), 50 mM DTT, 0.01% (w/v) bromophenol blue, pH 6.8) and sonicated (5 min). Cell samples were then separated using SDS–PAGE (12.5% (w/v) polyacrylamide), transferred to an activated nitrocellulose membrane (Bio-Rad), blocked with non-fat dry milk (Roth, 5% (w/v), TBS, 0.1% (v/v) Tween 20) and probed with an anti-acetyl-α-tubulin antibody (1:1,000, Sigma-Aldrich, T6793) and an anti-GAPDH antibody (1:2,000–1:5,000, Sigma-Aldrich, G9545) as a loading control (Fusion SL, peqlab). An uncropped blot is shown in Supplementary Fig. 8.

### Abundance of BubR1

HeLa cells plated in six-well plates were treated with SirReal2 dissolved in FCS (1% (v/v) DMSO, 16 h) at various concentrations. Cells were washed with PBS, lysed in IPLS (50 mM Tris/HCl, 150 mM NaCl, 0.5 mM EDTA, 0.5% (v/v) NP-40, pH 7.5, supplemented with Complete protease inhibitors (Roche)). Samples were pelleted and resuspended in 1 × SDS–PAGE sample buffer and heated (95 °C, 5 min). Cell samples were separated using SDS–PAGE (10% (w/v) polyacrylamide), transferred to a nitrocellulose membrane (Bio-Rad), blocked with non-fat dry milk (Roth, 5% (w/v), TBS, 0.1% (v/v) Tween 20) and probed with the anti-BubR1 (1:5,000, BD Biosciences, 612502) and anti-tubulin (1:5,000, Sigma-Aldrich, T5168) as a loading control. Uncropped blots are shown in the Supplementary Fig. 8.

### Immunocytochemistry

HeLa cells that were incubated with SirReal2 (20 and 50 μM), SirReal6 (50 μM), AGK2 (Sigma-Aldrich, 20 μM) or DMSO as a control in Dulbecco’s modified Eagle’s medium supplemented with 10% FCS, antibiotics and DMSO (1% (v/v)) for 4 h, were fixed with ice-cold methanol (10 min), washed with PBS and blocked with PBS supplemented with 0.1% (v/v) Triton-X-100 and 5% (v/v) FCS (30 min). Cells were then stained with an anti-acetyl-α-tubulin antibody (Sigma-Aldrich, T6793) and then probed with a secondary Alexa 546 conjugated anti-mouse-antibody (Invitrogen). Nuclei were counterstained with DAPI (4',6-diamidino-2-phenylindole). Coverslips were mounted with FluoroMount (Sigma-Aldrich) and sealed with DPX Mountant (Sigma-Aldrich). Images of the mounted samples were acquired on a Leica DM500 microscope equipped with a Leica DFC 395 FX camera and HBO 100 W lamp^40^. The microscope was run with the Leica Application Suite 4.4.0 software. Chroma UV filter set (No. C40888) and Leica N2.1 filter set (No. 513832) were used for DAPI and Alexa 546 signal acquisition, respectively, with a HCX FL Fluotar 40x/0.75 (dry) objective. Further details about the equipment and the settings that were used to acquire the images are found in the Supplementary Methods section. Unprocessed images are found in Supplementary Fig. 9.

### p53 Acetylation

U2OS cells (ATCC accession no. HTB-96) were seeded and cultured until they reached 90% confluency. Cells were then pretreated for 1 h with NCA (Sigma-Aldrich), SirReal2, SirReal5 or SirReal6 at the indicated concentrations, and then subsequently exposed to 20 J cm^−2^ ultraviolet light. Cells were incubated for an additional 6 h in the presence of the inhibitor and then lysed in IPLS (50 mM Tris/HCl, 150 mM NaCl; 0.5 mM EDTA; 0.5% (v/v) NP-40; 1 × Complete Protease Inhibitors (Roche), pH 8.0) and resuspended in 1 × Laemmli Buffer. Samples were then separated on SDS–PAGE, transferred to a nitrocellulose membrane (Bio-Rad), blocked with non-fat dry milk (Roth, 5% (w/v), TBS, 0.1% (v/v) Tween 20) and probed with anti-acetyl-p53 K382 (Cell Signaling, #2522), anti-p53 DO.1 (Santa Cruz Biotechnology, sc-126) and anti-vinculin (Cell Signaling, #4650) as a loading control. Uncropped blots are shown in the Supplementary Fig. 10.

## Author contributions

T.R., W.S., O.E. and M.J. designed the study and wrote the paper. T.R. performed the crystallization experiments, collected the data and analysed all the data. S.G. analysed the structural data. M.Schiedel synthesized SirReal inhibitors. C.R. performed the kinetic analysis. T.R., C.R., M.P., M.Schiedel and M.G. performed the inhibition tests. T.R., C.R., M. Schiedel and M. Schutkowski analysed *in vitro* data. B.J.N., T.R., K.S., K.I.L., A.L., J. Oláh, J. Ovádi and K.I.L. performed the cellular biology, T.R., B.J.N., A.L., J. Ovádi and D.A.S. analysed the cellular data. B.K. and W.S. performed computational analysis. All authors discussed and commented on the manuscript.

## Additional information

**Accession codes:** Coordinates and structure factors of the Sirt2-SirReal2-NAD^+^ complex (4RMG), Sirt2-SirReal2-H3 complex (4RMH), Sirt2-SirReal1-OTC complex (4RMI) and the Sirt2-ADPR-NCA (4RMJ) complex have been deposited in the Protein Data Bank under the above-mentioned accession codes.

**How to cite this article:** Rumpf, T. *et al.* Selective Sirt2 inhibition by ligand-induced rearrangement of the active site. *Nat. Commun.* 6:6263 doi: 10.1038/ncomms7263 (2015).

## Supplementary Material

Supplementary Information Supplementary Figure 1-10, Supplementary Tables 1-2, Supplementary Note 1, Supplementary Methods and Supplementary References

## Figures and Tables

**Table 1:** Data collection and refinement statistics.

	Sirt2–SirReal1–OTC*	Sirt2–SirReal2–H3*	**Sirt2–SirReal2–NAD**^+^*
*Data collection*			
Space group	*P*2_1_	*P*2_1_	*I*2
Cell dimensions (Å)			
*a*, *b*, *c* (Å)	36.21, 73.75, 55.86	35.99, 73.30, 55.29	83.7, 54.51, 96.69
α, β, γ (°)	90, 94.71, 90	90, 95.23, 90	90, 114.8, 90
Resolution (Å)†	44.43–1.45 (1.48–1.45)	44.02–1.42 (1.44–1.42)	48.28–1.88 (1.92–1.88)
*R*_merge_	0.126 (1.009)	0.060 (0.922)	0.068 (1.351)
*R*_pim_	0.054 (0.471)	0.035 (0.546)	0.029 (0.583)
*I*/*σI*	9.2 (1.7)	11.9 (1.5)	19.3 (1.5)
Completeness (%)	99.9 (99.9)	99.6 (99.6)	100 (100)
CC1/2	0.995 (0.549)	0.999 (0.534)	0.999 (0.585)
Redundancy	6.5 (6.2)	3.7 (3.8)	6.6 (6.4)
			
*Refinement*			
Resolution (Å)	44.43–1.45	44.02–1.42	48.28–1.88
No. of reflections	334,389 (16,809)	199,366 (10,043)	213,173 (12,962)
*R*_work_/*R*_free_ (%)	26.0/28.2	18.1/18.8	20.2/24.7
No. of atoms			
Protein	2,251	2,406	2,350
SirReal inhibitor	25	29	29
Ac-Lys peptide/NAD^+^	32	12	44
Zn^2+^	1	1	1
Water	156	242	99
*B*-factors (Å^2^)			
Protein	17.3	21.1	38.4
SirReal inhibitor	27.0	25.1	32.2
Ac-Lys peptide/NAD^+^	29.8	48.3	41.8
Zn^2+^	11.7	14.8	30.7
Water	20.5	27.0	36.0
r.m.s. deviations			
Bond lengths (Å)	0.012	0.008	0.014
Bond angles (°)	1.58	1.33	1.65

Ac-Lys, acetyl-lysine; r.m.s., root mean squared.

^*^Each data set was obtained from one single crystal. Sirt2–SirReal1–OTC and Sirt2–SirReal2–H3 were collected at 1.0 Å at the Swiss Light Source (Villigen, Switzerland), Sirt2–SirReal2–NAD^+^ was collected with an in-house X-ray source at 1.5418 Å.

^†^Values in parentheses are for highest-resolution shell.
