# Supplementary material for: Selective Sirt2 inhibition by ligand-induced rearrangement of the active site
Source: Nat Commun. 2015 Feb 12;6:6263. doi: 10.1038/ncomms7263 (PMC4339887; doi:10.1038/ncomms7263)
Supplement: Supplementary Information — Supplementary Figure 1-10, Supplementary Tables 1-2, Supplementary Note 1, Supplementary Methods and Supplementary References [file ncomms7263-s1.pdf]

SUPPLEMENTARY FIGURES

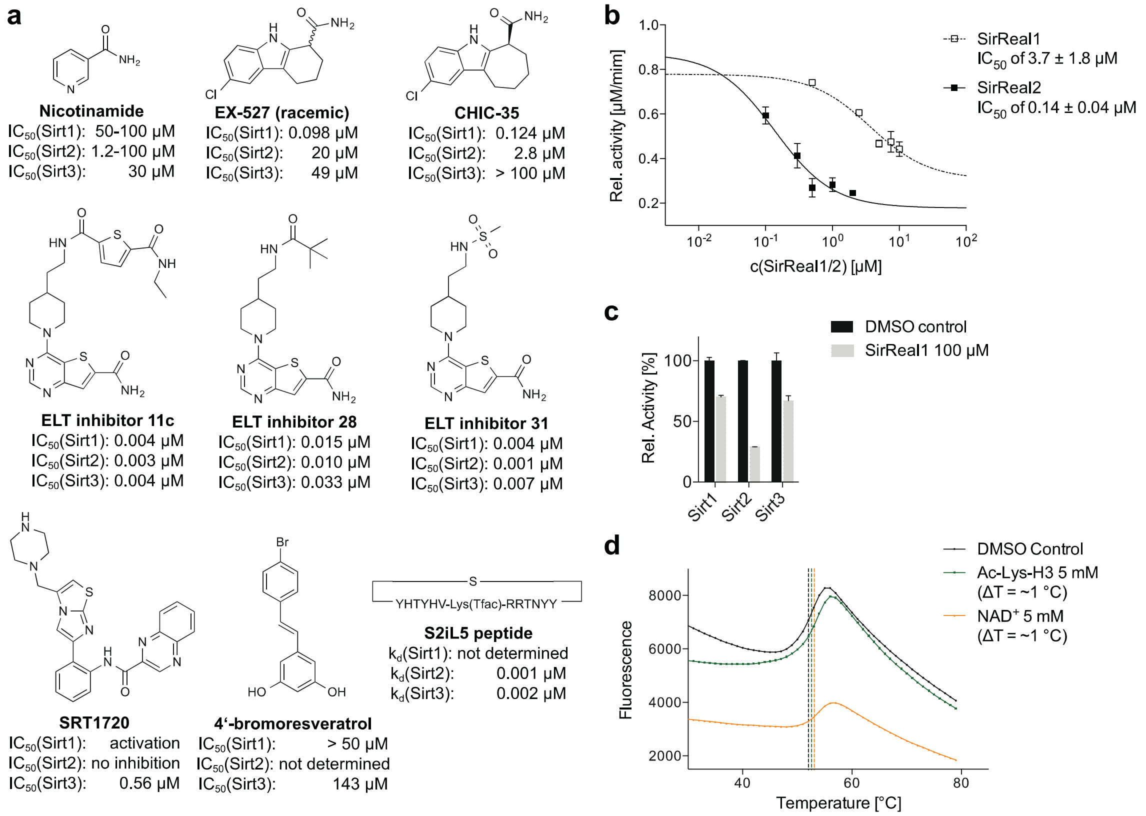

Supplementary Figure 1 Structures and inhibition data for sirtuin inhibitors with published structural information, *in vitro* inhibition data for SirReal1/2 and thermal stability plots for Sirt2. **(a)** Chemical structures and IC_{50} or k_d values of sirtuin inhibitors with known binding modes: nicotinamide[1], indoles (EX-527, CHIC-35)[2-4], ELT inhibitors[5], SRT1720[6,7], 4'-bromoresveratrol[8] and the macrocyclic peptide S2iL5[9]. **(b)** Dose-response curves for Sirt2 inhibition in an HPLC-based absorption assay in the presence of SirReal1/2 using a non-labeled acetyl-lysine oligopeptide based on α-tubulin. Data are presented as mean ± s.d. (n=2). **(c)** *In vitro* inhibition data for Sirt1-3 in the presence of SirReal1 (100 µM) using an HPLC-based absorption assay with a non-labeled acetyl-lysine oligopeptide based on α-tubulin as a substrate. A solution containing DMSO was used as a negative control. SirReal1 shows a preference to inhibit Sirt2 but also affects the activity of Sirt1/3 at higher concentrations. Data are presented as mean ± s.d.

(n=2). **(d)** Representative thermal stability plots of Sirt2 in the presence of either the cosubstrate NAD^+ or an acetyl-lysine oligopeptide. The presence of either NAD^+ or an acetyl-lysine oligopeptide only leads to a slight stabilization of Sirt2 as compared to a combination of SirReal2 and cosubstrate or the substrate, respectively (n=3, Fig. 1d).

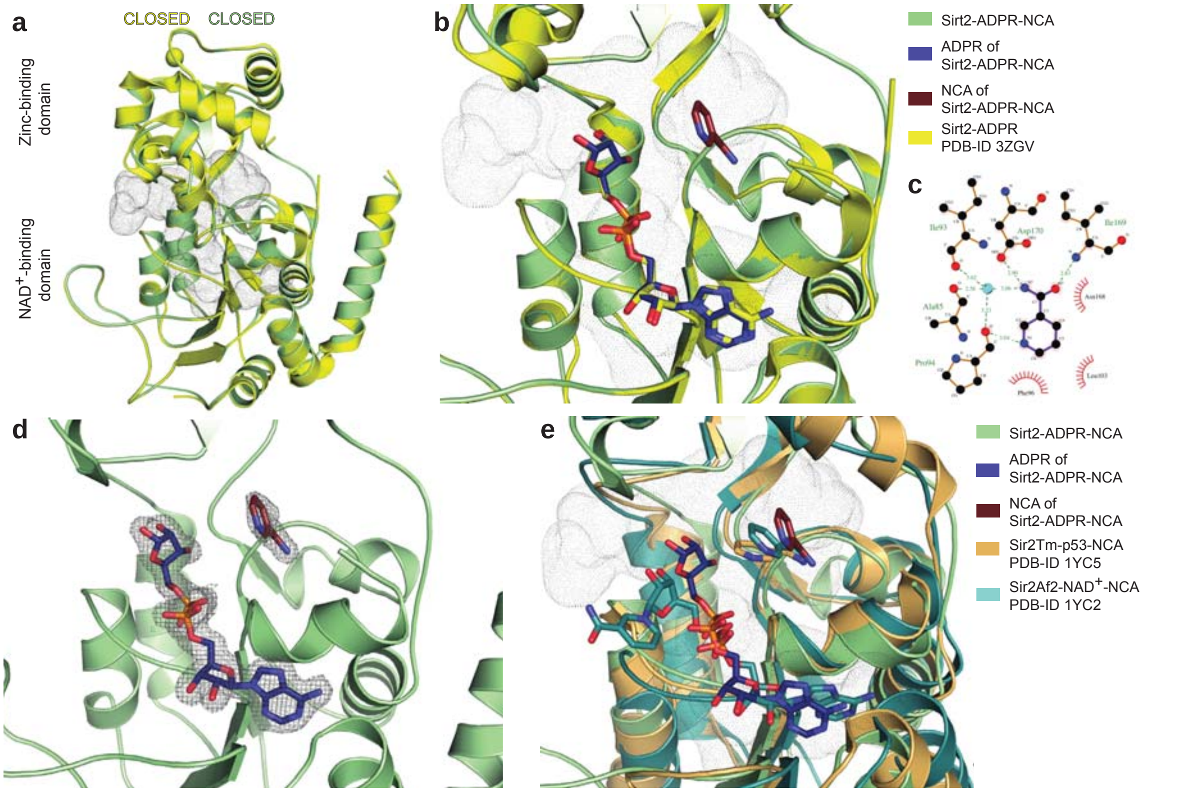

Supplementary Figure 2 Nicotinamide (NCA) of Sirt2-ADPR-NCA occupies the C-pocket and interacts over a network of highly conserved residues with Sirt2. **(a)** Superposition of the overall structure of Sirt2-ADPR-NCA (pale green) with the Sirt2-ADPR complex (PDB-ID 3ZGV[10], yellow). Both structures are very similar (r.m.s.d. (C_α atoms) = 0.44 Å) and resemble a 'closed' conformation, due to the binding of the Sirt2-specific insertion that acts as a pseudo-substrate for the neighboring Sirt2-molecule. The active site of Sirt2 is shown as small grey dots. Residues Tyr139-Gly141 of Sirt2-ADPR (PDB-ID 3ZGV) were not defined in the electron density map. **(b,d)** NCA (brown sticks) occupies the C-pocket in vicinity of ADPR (deep blue sticks). **(c)** The amide of the nicotinamide is tightly bound via hydrogen bonds to Ile169 and Asp170 while the pyridine ring undergoes π–π–stacking with Phe96. **(d)** Electron density maps of ADPR (overall B-factor 21.9 $Å^2$) and NCA (overall B-factor 46.3 $Å^2$) of Sirt2-ADPR-NCA. The position of the pyridine ring is more flexible relative to the amide as its B-factors are significantly higher than the B-factors of the amide (B-factor(Phenyl ring): 49.1 $Å^2$; B-factor(amide): 41.1 $Å^2$). The σ-weighted $2F_o$-F_c electron density map is contoured at 1.0 σ. A stereo image of **d** as well as a σ-

weighted F_o-F_c electron density OMIT map for ADPR and NCA are shown in Supplementary Fig. 4a,b. **(e)** The interactions of NCA with Sirt2 and its position within the C-pocket is similar to the ones observed in other sirtuin structures in complex with nicotinamide (Sir2Tm-Ac-Lys-p53-NCA, PDB-ID 1YC5[11], light orange, Sir2Af2-NAD$^+$-NCA, PDB-ID 1YC2[11], teal). However, in contrast to nicotinamide of Sir2Tm-Ac-Lys-p53-NCA and Sir2Af2-NAD$^+$-NCA the amide moiety and the phenyl ring of nicotinamide of Sirt2-ADPR-NCA do not lie in the same plane. The cofactor-binding loop in **b,d,e** is not shown for better clarity.

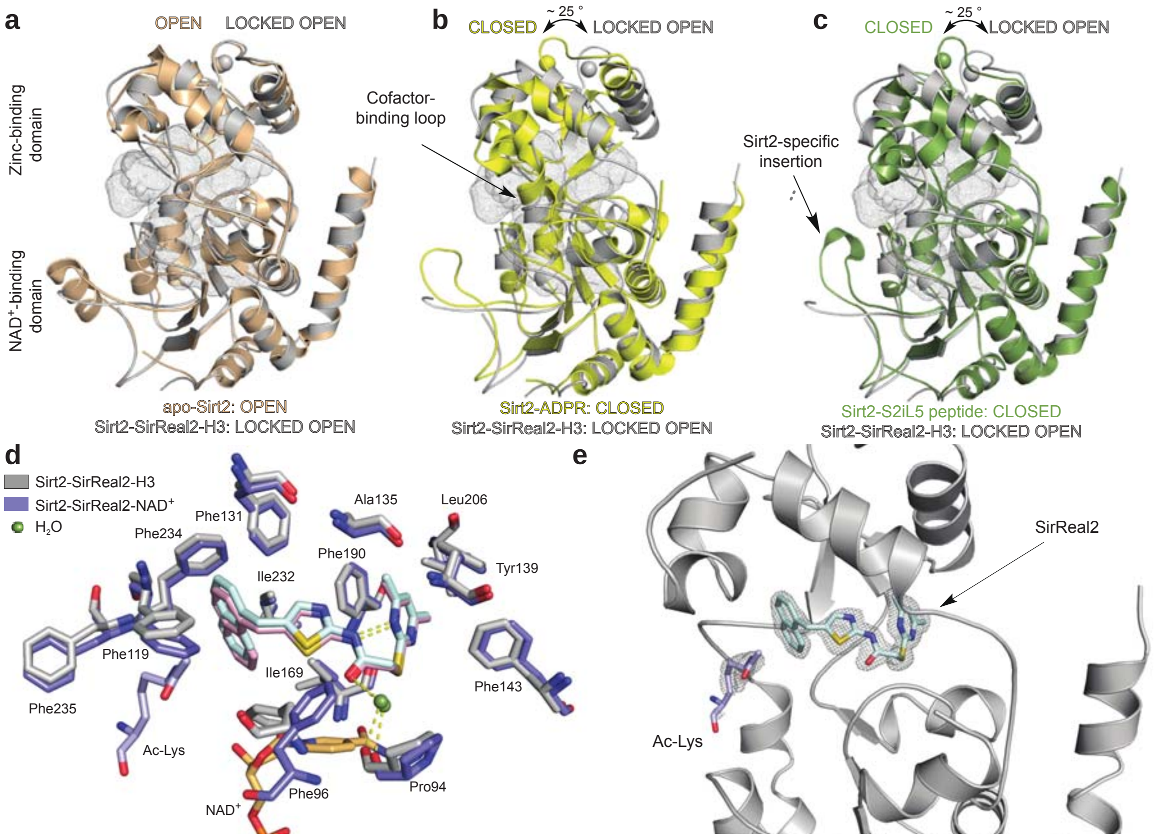

Supplementary Figure 3 SirReal2 functions as a molecular wedge and locks Sirt2 in an open conformation. **(a-c)** Superposition of Sirt2-SirReal2-H3 with Sirt2-apo (PDB-ID 3ZGO[10,12], salmon, residues 34-45 are omitted for better clarity), Sirt2 in complex with ADP ribose (PDB-ID 3ZGV, yellow, residues Tyr139-Gly141 were not defined in the electron density map) and the Sirt2-S2iL5-peptide complex (PDB-ID 4L3O[9], green). The active site is shown as small grey dots. As seen in Fig. 2 of the main article, the Sirt2-SirReal2 complex adopts an open state comparable to Sirt2-apo and in contrast to the Sirt2-ADPR and Sirt2-S2iL5 complex. Sirt2-SirReal2-H3 bears more resemblance to Sirt2-apo (r.m.s.d. (C_α atoms) = 1.3 Å) than to the Sirt2-ADPR complex (r.m.s.d. (C_α atoms) = 1.8 Å) or the Sirt2-S2iL5 peptide complex (r.m.s.d. (C_α atoms) = 1.6 Å). **(d)** Comparison of the interactions of SirReal2 in Sirt2-SirReal2-H3 (light cyan) with SirReal2 in Sirt2-SirReal2-NAD$^+$ (light pink). Interacting residues are represented as sticks (Sirt2-SirReal2-H3: light grey; Sirt2-SirReal2-NAD$^+$: slate blue). Binding of SirReal2 is almost identical (r.m.s.d. of 0.47 Å) in both structures. This is also true for the positions of the

SirReal2-interacting residues of the Sirt2-SirReal2 structures except for Phe96 which is forced to adopt a different position upon NAD^+-binding. Hydrogen bonds are shown as dashed yellow lines. **(e)** Electron density maps for SirReal2 (pale cyan sticks, overall *B*-factor of 25.1 $Å^2$) and the acetyl-lysine peptide substrate (light blue sticks, overall *B*-factor of 48.3 $Å^2$). The σ-weighted $2F_o$-F_c electron density map is contoured at 1.0 σ. A stereo image of **e** as well as a σ-weighted F_o-F_c electron density OMIT map for SirReal2 and the Ac-Lys-H3 oligopeptide is shown in Supplementary Fig. 5a,b.

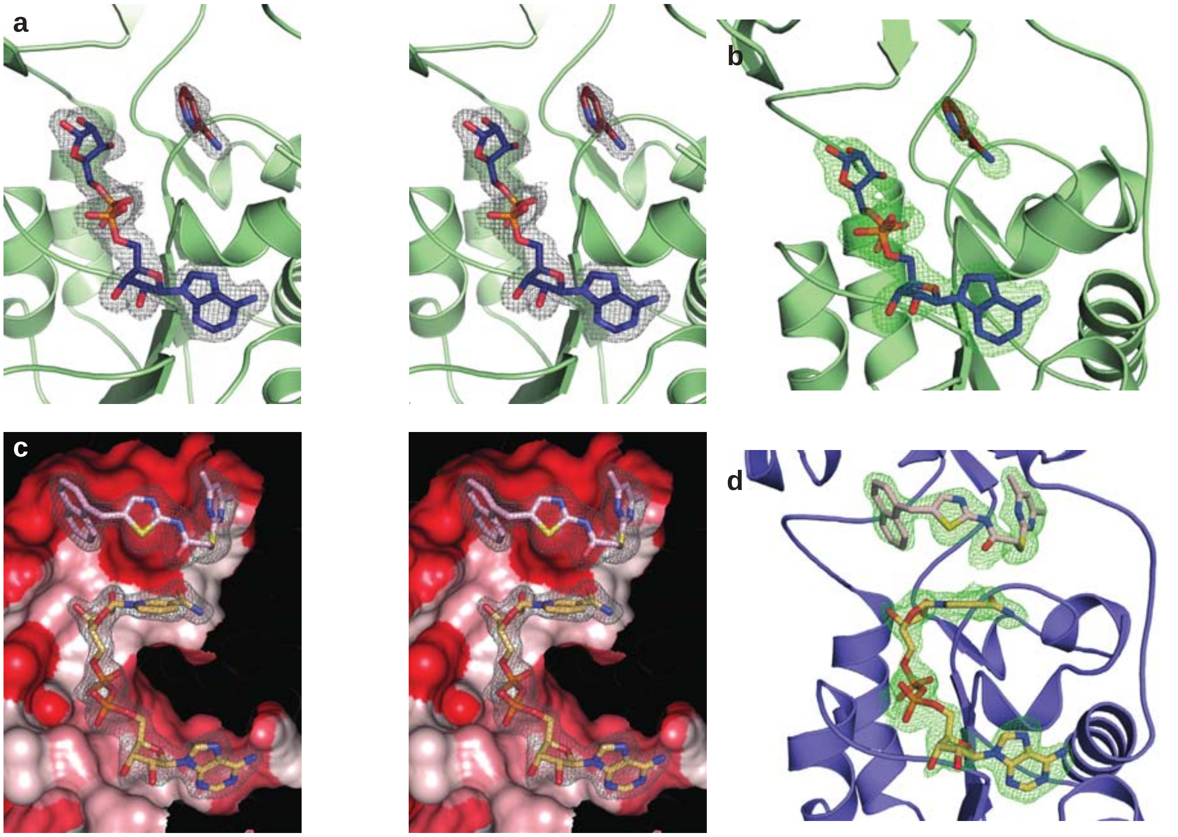

Supplementary Figure 4 Stereo images of the σ-weighted $2F_o$-F_c electron density and the σ-weighted F_o-F_c OMIT maps of presented inhibitors, cosubstrates and substrates. Wall-eyed stereo representation of the electron density of the ligands of **(a)** the Sirt2-ADPR-NCA structure and the Sirt2-SirReal2-NAD^+ complex **(c)**. Sirt2-ADPR-NCA is presented as a pale green cartoon and ADPR and NCA is shown as dark blue and dark brown sticks. Sirt2-SirReal2-NAD^+ is presented as a slate blue cartoon and SirReal2 and NAD^+ are shown as light pink and light orange sticks. The surface of Sirt2-SirReal2-NAD^+ of **c** is colored according to its hydrophobicity (red color indicating increasing hydrophobicity). The σ-weighted $2F_o$-F_c maps are contoured at 1.0 σ and shown as grey mesh . **(b,d)** The σ-weighted F_o-F_c electron density OMIT maps are contoured at 3.0 σ (ADPR, SirReal2) or 2.0 σ (NCA, NAD^+) and shown as green mesh. The cofactor-binding loop of **a-d** is omitted for clarity.

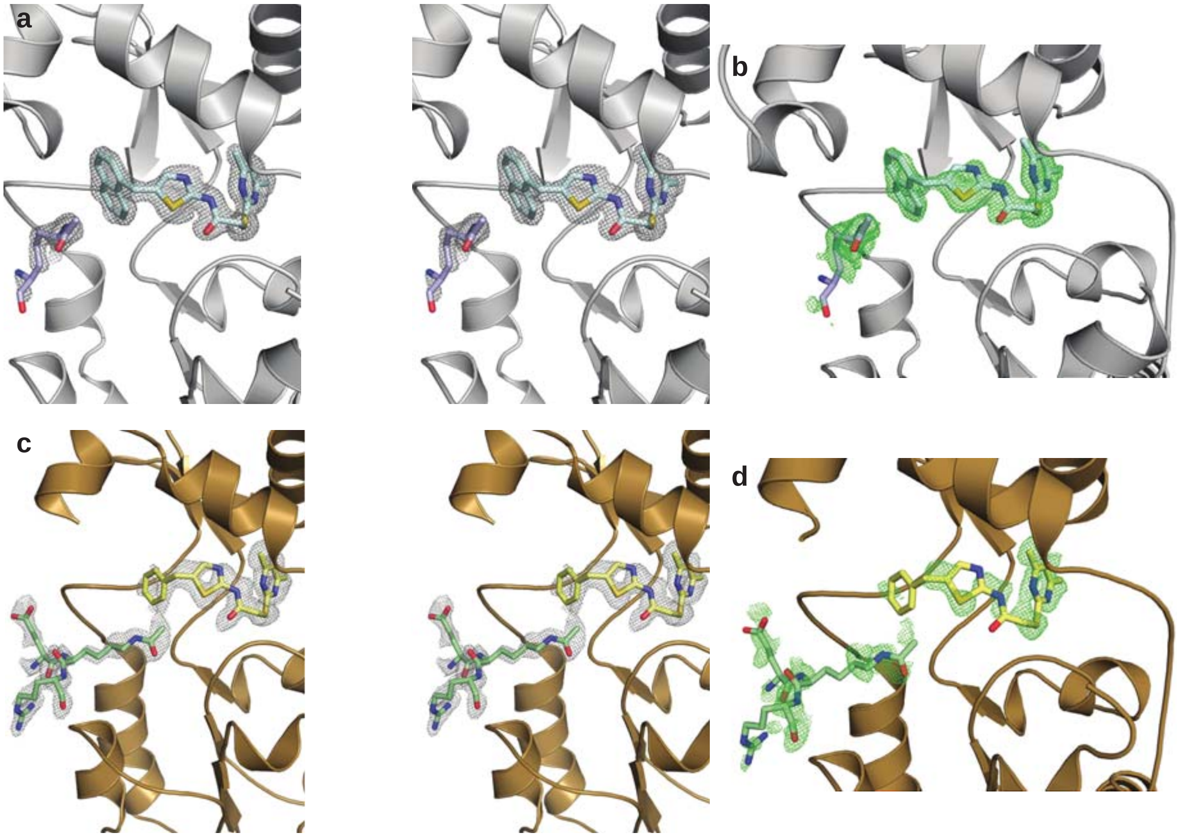

Supplementary Figure 5 Stereo images of the σ-weighted $2F_o$-F_c electron density and images of the σ-weighted F_o-F_c OMIT map of the Sirt2-SirReal2-H3 and Sirt2-SirReal1-OTC complexes. **(a,c)** Wall-eyed stereo representation of the electron density maps of the ligands of the Sirt2-SirReal2-H3 structure **(a)** and the Sirt2-SirReal1-OTC **(c)** complex. Sirt2-SirReal2-H3 is presented as a light grey cartoon and SirReal2 and the Ac-Lys-H3 oligopeptide are shown as light cyan and light blue sticks. Sirt2-SirReal1-OTC complex is presented as a brown cartoon and SirReal1 and the Ac-Lys-OTC oligopeptide are shown as pale yellow and light green sticks. The σ-weighted $2F_o$-F_c maps are contoured at 1.0 σ and shown as grey mesh. **(b,d)** The σ-weighted F_o-F_c electron density OMIT maps are contoured at 3.0 σ (SirReal2), 2.0 σ (Ac-Lys-H3, SirReal1) or 1.5 σ (Ac-Lys-OTC) and shown as green mesh. The poor electron density for the acetyl-lysine oligopeptides as well as the one of SirReal1 is probably due to a low occupancy. The cofactor-binding loop of **a-d** is omitted for clarity.

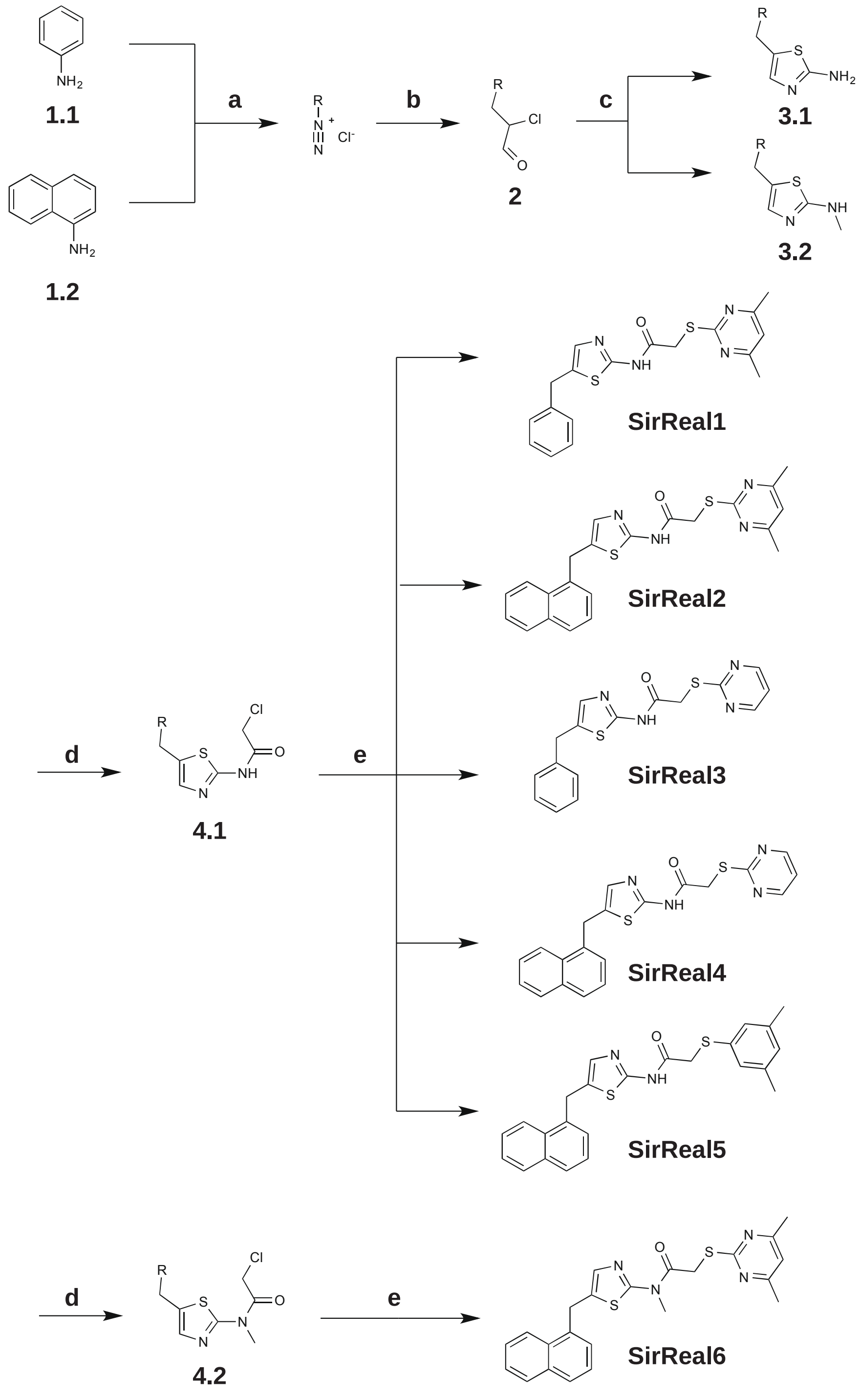

Supplementary Figure 6 Synthesis scheme for SirReal inhibitors. Preliminary tests with SirReal2 were performed with the commercially available compound (Chembridge, compound ID 7881488). For further characterization it was synthesized in our laboratory as the other SirReal inhibitors. Starting with the diazotization of aniline (**1.1**) for SirReal1/3 or

α-naphthylamine for SirReal2/4/5/6 (**1.2**), the α-chloropropanals (**2**) were generated via a Meerwein reaction[13,14]. Condensation of thiourea (SirReal1-5) or 1-methylthiourea (SirReal6) and the α-chloropropanals (**2**) led to formation of the aminothiazoles (**3.1 / 3.2**) which were then chloroacetylated to (**4.1 / 4.2**), followed by a nucleophilic substitution[15] with 2-mercapto-4,6-dimethylpyrimidine, 2-mercaptopyrimidine or 3,5-dimethylthiophenol to yield SirReal1-6. Identity and purity of all SirReal inhibitors were confirmed by ^{1}H-, ^{13}C-NMR, mass spectrometry respectively, and high performance liquid chromatography (HPLC) analysis. All synthesized SirReal inhibitors were of a purity of at least 95%. Spectroscopic data for all synthesized SirReal inhibitors can be found in the Supplementary Notes section. Reagents and conditions: (a) $NaNO_2$, HCl, water, 0 °C, 20 min; (b) acrolein, $CuCl_2 \times 2\,H_2O$, $NaHCO_3$, acetone, 20 °C, 3 h, 9% yield; (c) thiourea or 1-methylthiourea, ethanol, reflux, 2 h, 56% yield or 38%, respectively; (d) chloroacetyl chloride, DIPEA, acetonitrile, 20 °C, 2 h, 98% yield; (e) 2-mercaptopyrimidine, 2-mercapto-4,6-dimethylpyrimidine or 3,5-dimethylthiophenol, Na_2CO_3, KI, DMSO, 20 °C, 2 h, 80%, 89% or 13% yield.

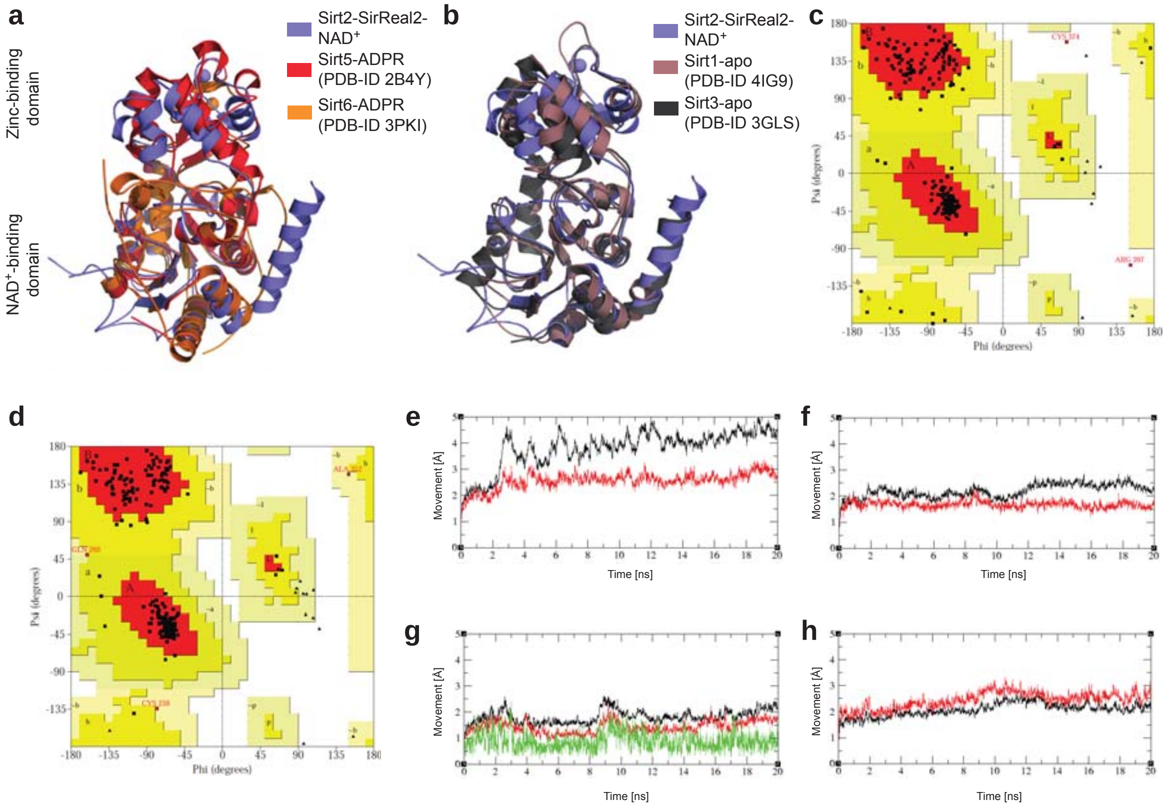

Supplementary Figure 7 Structural comparison of the Sirt2-SirReal2-NAD$^+$ complex with structures of available crystal structures of other sirtuins, stereochemical analysis, r.m.s.d. plots for molecular dynamics (MD) simulations of the Sirt1/3 homology models and the Sirt2-SirReal2-H3 structure. **(a,b)** Superposition of the Sirt2-SirReal2-NAD$^+$ complex with crystal structures of Sirt5 (red, PDB-ID 2B4Y[16]) and Sirt6 (orange, PDB-ID 3PKI[17]) in their open conformation **(a)** and with crystal structures of Sirt1 and Sirt3 (**b**, Sirt1-apo, brown, PDB-ID 4IG9[18]; Sirt3-apo, raspberry, PDB-ID 3GLS[19]). All structures adopt the 'open' conformation and show main differences in the conformation of the zinc-binding domain while the NAD$^+$-binding domain adopts a very similar conformation. The structural differences of the Sirt2-SirReal2-NAD$^+$ complex are more pronounced when compared to the crystal structures of Sirt5/6 than to the ones of Sirt1/3. This is also reflected by r.m.s.d. values: compared to Sirt5/6 (r.m.s.d. (C_α atoms) = 1.8–1.9 Å); to Sirt1/3 (r.m.s.d. (C_α atoms) = 1.6 Å). The C-terminal regulatory segment of Sirt1 is omitted for clarity. **(c)** PROCHECK stereochemical analysis of the Sirt1 homology model. 91.1% of the Φ and Ψ angles of the protein backbone are located in the most favored

regions, 8.1% are in the additional allowed regions and two residues are in the disallowed regions of the Ramachandran plot. Outlier residues are located outside of the binding pockets. **(d)** PROCHECK stereochemical analysis of the Sirt3 homology model. 92.2% of the Φ and Ψ angles of the protein backbone are located in the most favored regions, 6.5% are in the additional allowed regions and three residues are in the generously allowed regions of the Ramachandran plot. **(e-h)** r.m.s.d. plots for MD simulations of the Sirt1 homology model **(e)**, of the Sirt3 homology model **(f)**, of the Sirt2-SirReal2-H3 crystal structure with SirReal2 **(g)** and without SirReal2 **(h)**. The black line represents the r.m.s.d. value calculated for backbone heavy atoms (C_α, C and N) of the whole protein. The red line represents the r.m.s.d. value calculated for backbone heavy atoms of pocket residues only. The green line represents the r.m.s.d. value calculated for the heavy atoms of the inhibitor SirReal2.

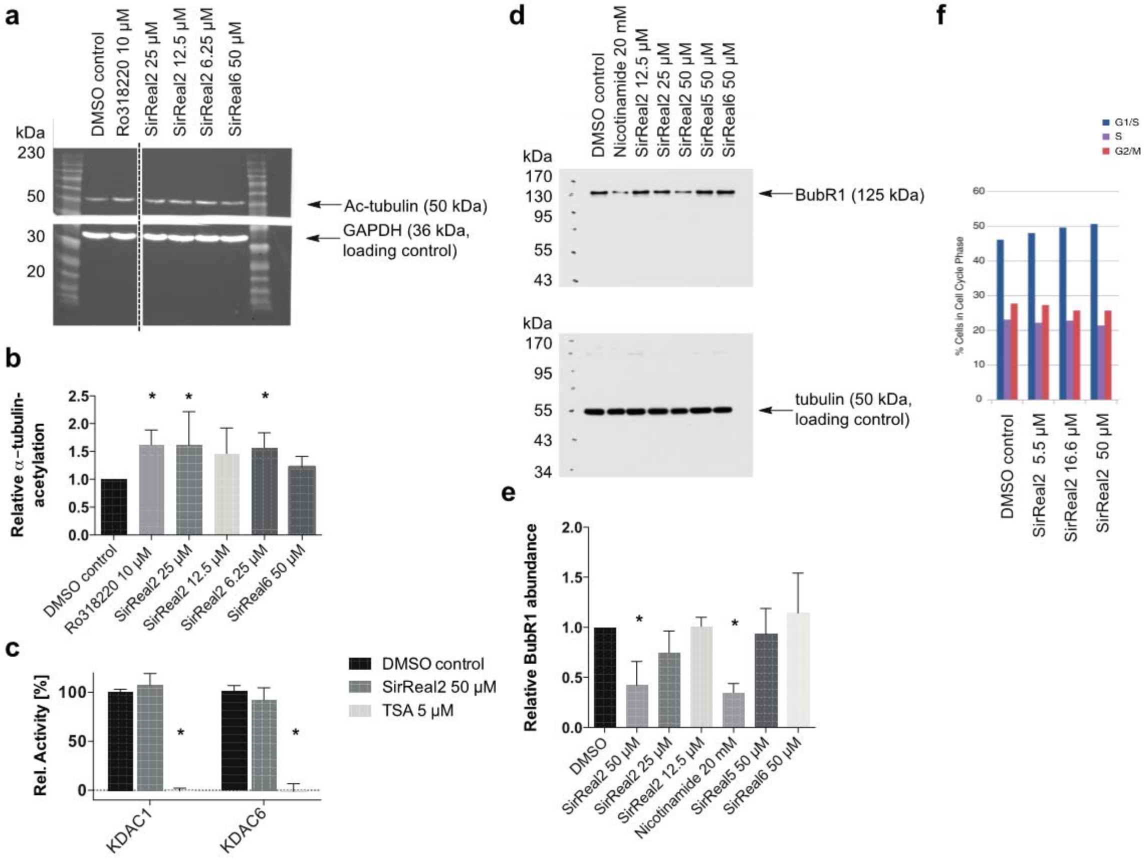

Supplementary Figure 8 SirReal2 induces a tubulin hyperacetylation and a significant depletion of BubR1. **(a)** Representative western blot of HeLa cell lysates after incubation with SirReal2 at the indicated concentrations. Ro318220 was used as a positive control[20]. The western blot was cut in half at around 40 kDa to detect acetyl-tubulin and GAPDH separately. **(b)** Quantification of the relative hyperacetylation after incubation with SirReal2. **(c)** *In vitro* inhibition data for the 'classical' KDAC1 and KDAC6. SirReal2 does not affect the activity of both KDACs. Only the positive control Trichostatin A (TSA) significantly inhibits both KDACs. **(d)** Representative western blot of HeLa lysates after incubation with SirReal2 at the indicated concentrations. SirReal2 induces a reduction of the spindle assembly checkpoint protein BubR1. **(e)** Statistical analysis of the abundance of BubR1 after incubation with SirReal2. At a SirReal2 concentration of 50 µM, BubR1 is significantly reduced in relation to the DMSO control. This is in line with a Sirt2-inhibition *in vivo*. **(f)** Cell cycle analysis after incubation of SirReal2 at the indicated concentrations. Treatment with SirReal2 did not alter the cell cycle. A description of

the cell cycle analysis can be found in the Supplementary Methods section. Colorplus™ Prestained Protein Ladder (New England Biolabs, tubulin hyperacetylation) and EZ-Run™ Prestained *Rec* Protein Ladder (Fisher Scientific, BubR1) were used as molecular weight markers. All data are presented as mean ± s.d. (n=3). *$P < 0.05$ as compared to the control.

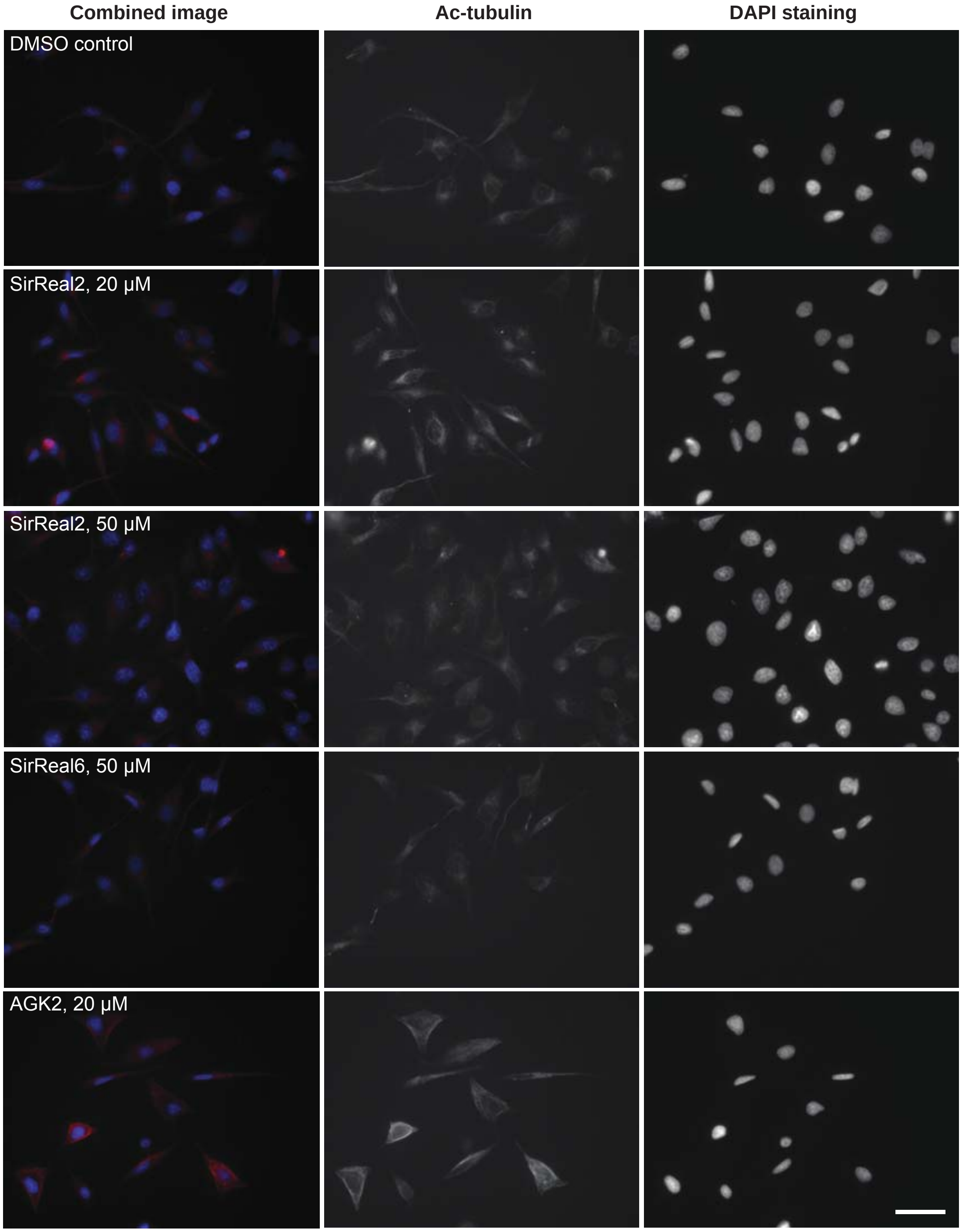

Supplementary Figure 9 SirReal2 induces tubulin-hyperacetylation in HeLa cells. Representative combined and raw images show that treatment with SirReal2 at a concentration of 20 µM and 50 µM induces hyperacetylation of the microtubule network as compared to the DMSO control. The effects are similar to the ones observed for the treatment with the Sirt2 inhibitor AGK2[21]. Treatment with SirReal6 on the other hand results in no substantial change of

acetylation level (n=4). The scale bar represents 10 μm .

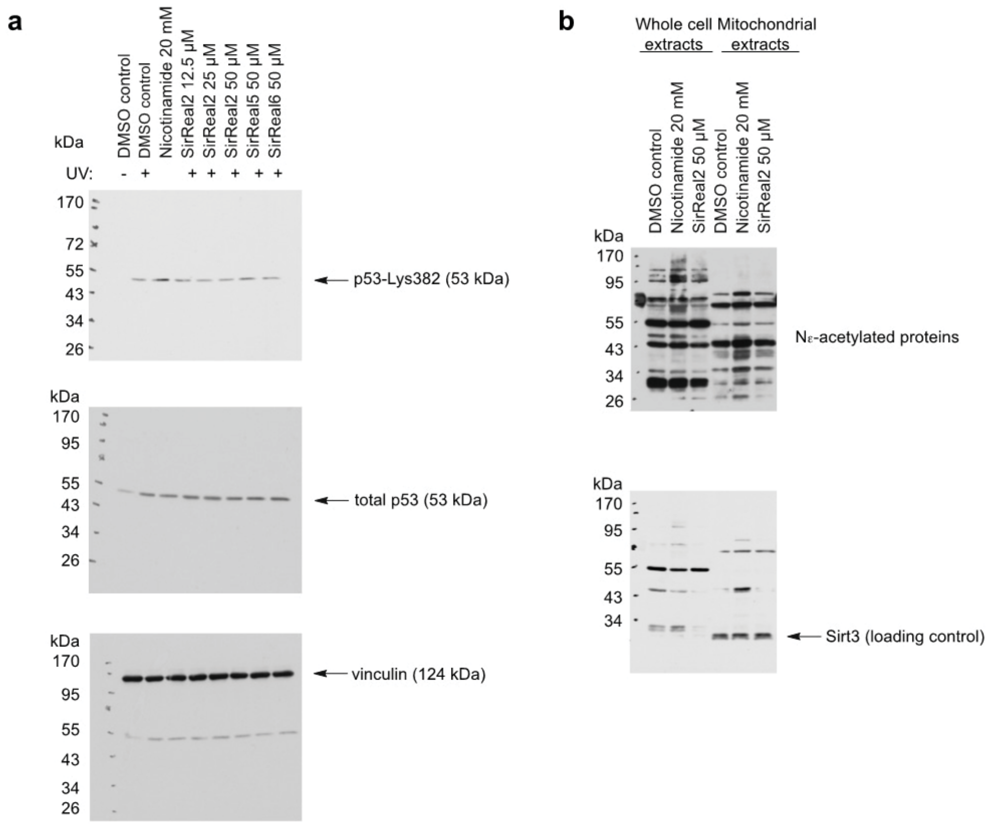

Supplementary Figure 10 SirReal2 does not alter the acetylation of p53-Lys382 or mitochondrial proteins. **(a)** Representative western blot of U2OS cell lysates after incubation with SirReal2. SirReal2 does not affect p53-Lys382-acetylation whereas treatment with nicotinamide, a pan-sirtuin inhibitor, results in an increase of p53-Lys382-acetylation (n=3). **(b)** Representative western blot of whole cell extracts and mitochondrial extracts of HEK293 cells. Treatment with SirReal2 does not alter the mitochondrial lysine-acetylation while incubation with the pan-sirtuin inhibitor nicotinamide leads to an increase of mitochondrial lysine acetylation. Sirt3 was used as a loading control. Sirt3 is enriched in the mitochondrial extracts as expected (n=3). EZ-Run™ Prestained *Rec* Protein Ladder (Fisher Scientific) was used as a molecular weight marker.

SUPPLEMENTARY TABLES

Supplementary Table 1 Physicochemical properties and selectivity profiles of sirtuin inhibitors (Supplementary Fig. 1).

	SirReal2	EX-527[2,3]	CHIC-35[2,4]	ELT inhibitor 11c[5]	ELT inhibitor 28[5]	ELT inhibitor 31[5]	SRT1720[6,7]	4'-bromo-resveratrol[8]	S2iL5[9]	Target values
IC_{50}/k_d for Sirt2 [µM]	0.14	20	2.4	0.003	0.01	0.001	n.d.*	n.d. *	0.001	
Selectivity<br>Sirt1/<br>Sirt2/<br>Sirt3 (fold)	 > 1,000<br>**1**<br>> 1,000	 **1**<br>200<br>500	 **1**<br>23<br>> 833	 1.3<br>**1**<br>1.3	 1.5<br>**1**<br>3.3	 4<br>**1**<br>7	 activation<br>n.d.*<br>**1**	 **1**<br>n.d.*<br>3†	 n.d.*<br>**1**<br>**1**	
Molar mass [g/mol]	420	249	263	487	389	383	470	291	2,039	< 500
log P[22]	4.63	2.76	3.1	2.18	0.78	2.76	2.62	4.13	0.4	< 5
H-donors	1	2	2	3	2	2	2	2	31	< 5
H-acceptors	4	1	1	6	5	6	5	0	23	< 10
Number of atoms	49	30	33	59	54	46	57	28	266	20-70

n.d. – not determined; † - inhibition substrate-dependent

Among sirtuin ligands with structural data available, SirReal2 presents the most potent and Sirt2-selective inhibitor. Its physicochemical properties as well as its selectivity make SirReal2 a suitable compound for studies on Sirt2 *in vivo.*

Supplementary Table 2: Refinement statistics for the Sirt2-ADPR-NCA complex structure.

	Sirt2-ADPR-NCA[b]
**Data collection**	
Space group	$P2_1\,2_1\,2_1$
Cell dimensions (Å)	
a, *b*, *c* (Å)	77.60, 77.96, 114.30
α, β, γ (°)	90, 90, 90
Resolution (Å)[a]	34.74–1.87 (1.91–1.87)
R_{merge}	0.123 (1.247)
R_{pim}	0.051 (0.530)
CC1/2	0.995 (0.639)
$I/\sigma I$	8.2 (1.3)
Completeness (%)	100 (100)
Redundancy	6.8 (6.5)
**Refinement**	
Resolution (Å)	34.74–1.87
No. reflections	393,009 (23,929)
R_{work} / R_{free} (%)	21.1 / 23.9
No. atoms	
Protein	4,754
ADP ribose	72
Nicotinamide	9
Zn^{2+}	2
Other entities	30
Water	138
B-factors (Å^2)	
Protein	42.6
ADP ribose	23.1
Nicotinamide	46.4
Zn^{2+}	54.6
Other entities	36.4
Water	34.5
R.m.s. deviations	
Bond lengths (Å)	0.012
Bond angles (°)	1.58

[a] Values in parentheses are for highest-resolution shell. [b] Data were obtained from one single crystal and were collected at 1.0 Å at the Swiss Light Source (Villigen, Switzerland).

SUPPLEMENTARY NOTE 1

COMPOUND CHARACTERIZATION DATA

SirReal1 - *N*-(5-Benzylthiazol-2-yl)-2-((4,6-dimethylpyrimidin-2-yl)thio)acetamide

^{1}H NMR (400 MHz, DMSO-D$_6$): δ 11.53 (bs, 1H), 7.34–7.28 (m, 2H), 7.26-7.20 (m, 3H), 7.12–7.10 (m, 1H), 6.85 (s, 1H), 4.08 (s, 2H), 3.93 (s, 2H), 2.53 (s, 6H); **^{13}C NMR** (100 MHz, DMSO-D$_6$): δ 169.72, 167.96, 167.20, 157.17, 139.34, 134.16, 132.43, 128.63, 128.39, 126.68, 116.85, 34.37, 32.98, 23.82; HRMS (m/z): $[M + Na]^+$ calcd. for $C_{18}H_{18}N_4NaOS_2$, 393.0814; found, 393.0815; overall yield 9%; isolated mass 24 mg.

SirReal2 - 2-((4,6 -Dimethylpyrimidin-2-yl)thio)-*N*-(5-(naphthalen-1-ylmethyl)thiazol-2-yl)acetamide

^{1}H NMR (400 MHz, CDCl$_3$): δ 11.45 (bs, 1H), 8.05–8.00 (m, 1H), 7.89–7.84 (m, 1H), 7.80–7.76 (m, 1H), 7.51–7.46 (m, 2H), 7.45–7.38 (m, 2H), 7.07 (s, 1H), 6.83 (s, 1H), 4.53 (s, 2H), 3.90 (s, 2H), 2.52 (s, 6H); **^{13}C NMR** (100 MHz, CDCl$_3$): δ 169.79, 167.95, 167.11, 156.63, 135.15, 134.92, 133.91, 132.14, 131.50, 128.73, 127.71, 126.73, 126.15, 125.66, 125.51, 123.61, 116.82, 34.31, 30.43, 23.78; HRMS (m/z): $[M + Na]^+$ calcd. for $C_{22}H_{20}N_4NaOS_2$, 443.0971; found, 443.0973; overall yield 5%; isolated mass 45 mg.

SirReal3 - *N*-(5-Benzylthiazol-2-yl)-2-(pyrimidin-2-ylthio)acetamide

^{1}H NMR (400 MHz, DMSO-D$_6$): δ 12.23 (bs, 1H), 8.58 (d, *J* = 4.8 Hz, 2H), 8.17–8.12 (m, 1H), 7.96–7.90 (m, 1H), 7.86–7.80 (m, 1H), 7.58–7.43 (m, 4H), 7.31 (s, 1H); 7.17 (t, *J* = 4.8 Hz, 1H), 4.55 (s, 2H), 4.12 (s, 2H); **^{13}C NMR** (100 MHz, DMSO-D$_6$): δ 170.38, 166.67, 158.21, 156.94, 136.56, 135.13, 133.93, 131.84, 131.42, 129.01, 127.77, 127.02, 126.63, 126.23, 126.11, 124.28, 117.89, 34.46, 29.82; HRMS (m/z): $[M + Na]^+$ calcd. for $C_{20}H_{16}N_4NaOS_2$, 415.0659; found, 415.0659; overall yield 18%; isolated mass 102 mg.

SirReal4 - *N*-(5-(Naphthalen-1-ylmethyl)thiazol-2-yl)-2-(pyrimidin-2-ylthio)acetamide

^{1}H NMR (400 MHz, $CDCl_3$): δ 11.00 (bs, 1H), 8.66 (d, *J* = 4.9 Hz, 2H), 7.34–7.29 (m, 2H), 7.26–7.21 (m, 3H), 7.16–7.14 (m, 1H), 7.12 (t, *J* = 4.9 Hz, 1H), 4.08 (s, 2H), 3.99 (s, 2H); **^{13}C NMR** (100 MHz, $CDCl_3$): δ 170.64, 166.73, 157.81, 157.25, 139.09, 133.38, 132.78, 128.70, 128.40, 126.80, 117.58, 34.35, 32.96; HRMS (m/z): $[M + Na]^+$ calcd. for $C_{16}H_{14}N_4NaOS_2$, 365.0501; found, 365.0503; overall yield 11%; isolated mass 24 mg.

SirReal5 - 2-((3,5 -Dimethylphenyl)thio)-*N*-(5-(naphthalen-1-ylmethyl)thiazol-2-yl)acet-amide

^{1}H-NMR (400 MHz, DMSO-D_6): δ 12.13 (bs, 1H), 8.18–8.08 (m, 1H), 7.97–7.89 (m, 1H), 7.87–7.80 (m, 1H), 7.57–7.42 (m, 4H), 7.29 (s, 1H), 6.95 (s, 2H), 6.80 (s, 1H), 4.55 (s, 2H), 3.83 (s, 2H), 2.17 (s, 6H); **^{13}C-NMR** (100 MHz, DMSO-D_6): δ 167.38, 156.69, 138.56, 136.50, 135.20, 135.00, 133.95, 132.00, 131.44, 129.02, 128.37, 127.79, 127.02, 126.64, 126.44, 126.23, 126.11, 124.26, 36.31, 29.82, 21.16; HRMS (m/z): $[M + Na]^+$ calcd. for $C_{24}H_{22}N_2NaOS_2$, 441.1066; found, 441.1068; overall yield 2%; isolated mass 9 mg.

SirReal6 - 2-((4,6-Dimethylpyrimidin-2-yl)thio)-*N*-methyl-*N*-(5-(naphthalen-1-ylmethyl)-thiazol-2-yl)acetamide

^{1}H-NMR (400 MHz, DMSO-D_6,): δ 8.16–8.09 (m, 1H), 7.96–7.89 (m, 1H), 7.86–7.79 (m, 1H), 7.56–7.42 (m, 4H), 7.38 (s, 1H), 6.92 (s, 1H), 4.55 (s, 2H), 4.36 (s, 2H), 3.74 (s, 3H), 2.25 (s, 6H); **^{13}C-NMR** (100 MHz, DMSO-D_6): δ 169.23, 168.57, 167.38, 158.54, 136.50, 134.48, 133.94, 133.23, 131.45, 129.00, 127.77, 127.00, 126.59, 126.20, 126.07, 124.28, 116.48, 34.94, 34.36, 29.79, 23.65; HRMS (m/z): $[M + Na]^+$ calcd. for $C_{23}H_{22}N_4NaOS_2$, 457.1127; found, 457.1128; overall yield 21%; isolated mass 92 mg.

SUPPLEMENTARY METHODS

Homology Modeling . Homology models of human $Sirt1_{241–512}$ (UniProt: Q96EB6) and human $Sirt3_{122–395}$ (Uniprot: Q9NTG7) were generated using the Sirt2-SirReal2-H3 and Sirt2-SirReal2-NAD^+ crystal structures as templates. 10 protein conformations were generated and evaluated with the Modeller program version 9.11[23]. The model showing the most favorable DOPE (Discrete Optimized Protein Energy) assessment score[24] was selected. The stereochemical quality of the models was validated with PROCHECK[25] (Supplementary Fig. 6). All parameters indicate high-quality model structures. Assignment of correct protonation state and energy minimization of the homology models were carried out in MOE 2012.102[26].

Molecular dynamics s imulations. Molecular dynamics (MD) simulations were carried out for the Sirt2-SirReal2-H3 X-ray structure in uncomplexed form (inhibitor and substrate deleted) and in complex with SirReal2 (substrate deleted) as well as for the Sirt1 and Sirt3 homology models (uncomplexed) using the program AMBER 12 and the AMBER 2003 force field[27]. Missing residues in the Sirt2 structures were modelled using the loop search module of Modeller. Atom types and AM1-BCC atomic charges[28] were generated for the ligand using the Antechamber module. Ligand parameters were obtained from the general AMBER force field GAFF[29]. Preparation of the ligand-protein complex, addition of counter ions, solvation, preparation of parameter/topology and coordinate files was carried out using the LEaP module in AMBER. Parameters and libraries for zinc binding residues were defined as previously described[30].

The system was solvated using the water model TIP3BOX[31] and a margin of 10 Å. Two consecutive steps of minimization were carried out. In the first step 3,000 iterations (first 1,000 steepest descent and then 2,000 conjugate gradient) and in the second step 4,000 iterations (first 2,000 steepest descent and then 2,000 conjugate gradient) were applied to the

system. In the first step, atom coordinates for the amino acid residues and ligand atoms were restrained to their initial coordinates with a force constant of 500 kcal $mol^{-1}Å^{-2}$ to relieve the unfavorable van-der-Waals contacts in the surrounding solvent and thus to minimize the positions of the water molecules and ions. In the second step, restraints to atoms were removed and the whole system was minimized freely to relieve bad contacts in the entire system.

The temperature of the system was then equilibrated at 300 K through 100 ps of MD with a time step of 2 fs per step. A constant volume periodic boundary was set to equilibrate the temperature of the system by the Langevin dynamics[32] using a collision frequency of 1 ps^{-1} during the temperature equilibration routine. The protein and ligand atoms were restrained to the initial coordinates with a weak force constant of 10 kcal $mol^{-1}Å^{-2}$. The final coordinates obtained after temperature equilibration step were then used for a 20 ns MD routine during which the temperature was kept at 300 K by the Langevin dynamics using a collision frequency of 1 ps^{-1}. Constant pressure periodic boundary was used to maintain the pressure of the system at 1 bar using isotropic pressure scaling with a relaxation time of 2 ps. During the temperature equilibration and MD routines a non-bonded cut-off distance of 10 Å was used applying the Particle Mesh Ewald (PME) method[33] for calculating the full electrostatic energy of the periodic system and the SHAKE algorithm[34] to adjust the constraints of all bonds involving hydrogen.

Docking studies . All protein structures were prepared by using the Structure Preparation module in MOE 2012.102[26]. Hydrogen atoms were added, for titratable amino acids the protonation state was calculated using the Protonate 3D module in MOE. All protein structures were energy minimized using the AMBER99 force field using a tethering force constant of 1.5 kT σ^{-2} with (σ = 0.5 Å) for all atoms during the minimization. Water molecules and ligand atoms except the zinc ion were removed from the structures. Docking

studies were performed using the Glide program (Schrödinger Suite 2012-5.8)[35]. All sirtuin structures were superimposed on their backbone atoms using the Superpose module in MOE 2012.102[26]. The position of the inhibitor SirReal2 in the crystal structure was used to define the binding site (10 Å radius). 20 docking poses were calculated for each ligand. All other options were left at their default values. The best-ranked pose from each docking run was included in the analysis and visually inspected together with the protein structure using the program MOE 2012.102[26]. The applied docking protocol used in Glide was able to correctly reproduce the location and conformation of the inhibitor SirReal2 in the corresponding X-ray structures (SirReal2: 0.32 Å and 0.31 Å, respectively). In case of the generated homology models of human Sirt1 and Sirt3 the docking protocol gave docking poses with less favorable Glide SP scores and larger deviations from the conformation and location observed in the Sirt2-SirReal2-H3 structure. To compare the three sirtuins we selected for both homology models docking poses that showed a comparable interaction with the residues of the extended C-site and the selectivity pocket. Docking of SirReal2 to the available crystal structures of Sirt1 and Sirt3 (apo-form and different inhibitor-complexes) was not possible due to the limited size of the extended C-site and the selectivity pocket (data not shown).

Synthesis of α-tubulin derivative H-PSDK(Ac)TIGGWW-NH_2. The peptide (residues 36–44 of α-tubulin with two additional C-terminal tryptophans) was synthesized with standard solid-phase-peptide synthesis using 9-fluorenylmethoxy-carbonyl (Fmoc) amino acids. The Rink amide MBHA resin was incubated with *N*,*N*-dimethylformamide (DMF) at RT for 20 min while stirring. The Fmoc group was removed by incubation with 20% (vol/vol) piperidine in DMF at RT for 15 min under stirring. After washing with DMF (5 min, 5 times) the resin was incubated with 4 equivalents (eq) of amino acid, 4 eq *N*,*N*,*N′*,*N′*-tetramethyl-*O*-(1*H*-benzotriazol-1-yl)uronium hexafluorophosphate (HBTU) and 8 eq *N*,*N*-diisopropylethylamine (DIPEA) in DMF at RT for 45 min under stirring. After washing the resin with DMF (5 min,

5 times), Fmoc deprotection and amino acid coupling were repeated until the last amino acid was coupled and the Fmoc group was removed. The resin was washed with dichloromethane (DCM, 3 min, 5 times), methanol (5 min, 3 times) and DCM (3 min, 5 times). The peptide was cleaved from the resin and deprotected by incubation with 97% (vol/vol) trifluoroacetic acid (TFA) at RT (1 h, 2 times). Cleaved peptide was precipitated with cold diethylether, filtrated and dried. The peptide was purified by semi-preparative HPLC (Merck-Hitachi High Speed LC system) using a Merck Hibar LiChrosorb RP-8 column (250–25 mm, 7 µm). For separation a linear gradient from 20–50% (vol/vol) acetonitrile (ACN) with 0.1% (vol/vol) TFA in 60 min was applied (flow-rate: 8 ml min^{-1}). The purification yielded > 97% of pure peptide. Identity was confirmed by MALDI-MS (calculated mass: 1186.5 Da, found: 1187.5 Da).

***In vitro* KDAC1/6 assay**. Inhibition tests with SirReal2 and KDAC1/6 were conducted with a high-throughput fluorescence-based assay using the substrate ZMAL (Z-Lys(Acetyl)-AMC)[36]. ZMAL (12.6 µM final concentration) was mixed with assay buffer (50 mM Tris/HCl, 137 mM NaCl, 2.7 mM KCl, 1 mM $MgCl_2$, 0.1 mg mL^{-1} BSA, 5–10% (vol/vol) DMSO, 50 µL, pH 8.0) and SirReal2. A solution that contained DMSO was used a negative control. A solution with trichostatin A (TSA, Enzo Life Sciences) was used a positive control. The reaction was started through the addition of KDAC1 (Enzo Life Sciences, 10 µL) or KDAC6 (Enzo Life Sciences, 10 µL) and incubated (37 °C, 90 min, 150 rpm). To assure initial state conditions the substrate conversion was adjusted to 10–30% prior to inhibition tests. The reaction was stopped by the addition of a solution containing trypsin and TSA (50 mM Tris/HCl, 100 mM NaCl, 0.2% (vol/vol) DMSO, trypsin 5.5 U μL^{-1}, 16.5 µM TSA, pH 8.0, 60 µL) and further incubated (37 °C, 20 min, 150 rpm). Then fluorescence intensity of the released fluorophore of the deacetylated lysine derivative was measured in a microplate

reader (BMG Polarstar, λ_{ex} 390 nm, λ_{em} 460 nm). The amount of inhibition was determined with respect to the mixture with only DMSO.

Immunocytochemistry equipment and settings. The signal of Alexa 546 and DAPI of the same region were acquired with constant illumination parameters as grayscale 8-bit 600 dpi tiff files (1600×1200 pixels). Alexa 546 was acquired after 639.1 ms with a gamma factor of 0.65 without multiplying. The gain was set to 1. DAPI-stained images were acquired after about 20 ms with the same parameters as the one for Alexa 546 image acquisition. The temperature in the microscope room was kept at RT. Representative regions of each samples were processed with Adobe Photoshop CS2 to generate images in RGB mode by copying the appropriate original greyscale images to the blue (nuclei, DAPI) and the red (acetylated α-tubulin signal, Alexa 546) channel, respectively. As adjustment, a minimal background subtraction was applied on all images during processing in the same manner. The resolution was changed from 600 dpi (source images) to 450 dpi without resampling (Fig. 7 of the main article). LUT (CLUT) of grayscale images of the acetylated α-tubulin signal and of the DAPI signal are shown in Supplementary Fig. 8.

Cell Cycle Analysis . HeLa cells were treated as described in the Abundance of BubR1 section of the main article. Cells were harvested by trypsinization, washed in PBS, and fixed in ice-cold 70% (vol/vol) ethanol, and stored at –20 ºC for 2 h. Fixed cells were pelleted, washed in PBS, and stained in propidium iodide buffer (0.1% (wt/vol) sodium citrate, 0.3% (vol/vol) Triton X-100, 0.01% (wt/vol) propidium iodide, 0.02 mg mL^{-1} RNase A) as described previously[37] for 30 min. Stained cells were subjected to flow cytometric analysis using a FACSCaliber (BD Biosciences) and analyzed by FlowJo software (Tree Star, Inc.).

Cell cultivation. HEK-293 cells (ATCC accession no. CRL-1573) were grown in Dulbecco's modified Eagle's medium (DMEM, PAA) containing 10% (vol/vol) fetal calf serum (FCS, PAA), 1% (vol/vol) penicillin (PAA), 1% (vol/vol) streptomycin (PAA), 1% (vol/vol), L-glutamine (PAA) at 37 °C in a 5% (vol/vol) CO_2 atmosphere.

Mitochondrial extra ction. HEK-293 cells (ATCC accession no. CRL-1573) were treated with nicotinamide or SirReal2 at the indicated concentrations for 16 h. Cells were washed in PBS and lysed in 5 packed cell volumes of lysis buffer (20 mM HEPES/KOH, 250 mM sucrose; 10 mM KCl, 1.5 mM $MgCl_2$, 1 mM EDTA, 1 mM EGTA, 1 mM DTT; 0.1 mM PMSF, 1×Complete Protease Inhibitor Cocktail (Roche), pH 7.5). Cells were then homogenized in a Dounce homogenizer and 10% (vol/vol) of sample was removed for use as whole cell extract (WCE). To the remaining lysate, unbroken cells and nuclei were removed by centrifugation (10 min, 800*g*). The supernatant was transferred and centrifuged (10 min, 7,000*g*) to pellet mitochondria. Mitochondria were washed twice in lysis buffer, repeating centrifugation after each wash, followed by resuspending washed mitochondria in lysis buffer. NP-40 was added to both whole cell extracts and mitochondria fractions to a final concentration of 0.5% (vol/vol) and lysates were incubated at 4°C for 30 minutes. Lysates were cleared by centrifugation, protein concentrations were quantitated and normalized with 1×Laemmli Buffer. Whole cell and mitochondrial extracts were separated on SDS-PAGE and transferred to a nitrocellulose membrane (Bio-Rad), blocked with non-fat dry milk (Roth, 5% (wt/vol), TBS, 0.1% (vol/vol) Tween 20), and probed with anti-acetyl-lysine (Cell Signaling, #9814) and anti-Sirt3 (Cell Signaling, #2627) as a control for mitochondria enrichment and loading.

Statistical analysis . Statistical analysis for the BubR1 abundance and hyperacetylation of α-tubulin were performed with *t*-test option of Microsoft Excel using a one-tailed distribution.

For that the intensity of the bands were quantified (Fusion SL, peqlab) and normalized to the respective loading control.

SUPPLEMENTARY REFERENCES

1. Lawson, M. *et al.* Inhibitors to understand molecular mechanisms of NAD(+)-dependent deacetylases (sirtuins). *Biochim. Biophys. Acta* **1799,** 726–739 (2010).
2. Napper, A. D. *et al.* Discovery of indoles as potent and selective inhibitors of the deacetylase SIRT1. *J. Med. Chem.* **48,** 8045–8054 (2005).
3. Gertz, M. *et al.* Ex-527 inhibits Sirtuins by exploiting their unique NAD+-dependent deacetylation mechanism. *Proc. Natl. Acad. Sci.* **110,** E2772–E2781 (2013).
4. Zhao, X. *et al.* The 2.5 Å crystal structure of the SIRT1 catalytic domain bound to nicotinamide adenine dinucleotide (NAD+) and an indole (EX527 analogue) reveals a novel mechanism of histone deacetylase inhibition. *J. Med. Chem.* **56,** 963–969 (2013).
5. Disch, J. S. *et al.* Discovery of thieno[3,2-d]pyrimidine-6-carboxamides as potent inhibitors of SIRT1, SIRT2, and SIRT3. *J. Med. Chem.* **56,** 3666–3679 (2013).
6. Nguyen, G. T. T., Schaefer, S., Gertz, M., Weyand, M. & Steegborn, C. Structures of human sirtuin 3 complexes with ADP-ribose and with carba-NAD(+) and SRT1720: binding details and inhibition mechanism. *Acta Crystallogr. D Biol. Crystallogr.* **69,** 1423–1432 (2013).
7. Jin, L. *et al.* Biochemical characterization, localization, and tissue distribution of the longer form of mouse SIRT3. *Protein Sci.* **18,** 514–525 (2009).
8. Nguyen, G. T. T., Gertz, M. & Steegborn, C. Crystal structures of sirt3 complexes with 4'-bromo-resveratrol reveal binding sites and inhibition mechanism. *Chem. Biol.* **20,** 1375–1385 (2013).
9. Yamagata, K. *et al.* Structural Basis for Potent Inhibition of SIRT2 Deacetylase by a Macrocyclic Peptide Inducing Dynamic Structural Change. *Structure* **22,** 345–352 (2014).
10. Moniot, S., Schutkowski, M. & Steegborn, C. Crystal structure analysis of human Sirt2 and its ADP-ribose complex. *J. Struct. Biol.* **182,** 136–143 (2013).
11. Avalos, J. L., Bever, K. M. & Wolberger, C. Mechanism of sirtuin inhibition by nicotinamide: altering the NAD(+) cosubstrate specificity of a Sir2 enzyme. *Mol. Cell* **17,** 855–868 (2005).
12. Finnin, M. S., Donigian, J. R. & Pavletich, N. P. Structure of the histone deacetylase SIRT2. *Nat. Struct. Biol.* **8,** 621–625 (2001).
13. Krasavin, M. *et al.* Discovery and potency optimization of 2-amino-5-arylmethyl-1,3-thiazole derivatives as potential therapeutic agents for prostate cancer. *Arch. Pharm.*

342, 420–427 (2009).

14. Obushak, N. D., Matiichuk, V. S., Vasylyshin, R. Y. & Ostapyuk, Y. V. Heterocyclic Syntheses on the Basis of Arylation Products of Unsaturated Compounds: X. 3-Aryl-2-chloropropanals as Reagents for the Synthesis of 2-Amino-1,3-thiazole Derivatives. *Russ. J. Org. Chem.* **40,** 383–389 (2004).
15. Zav'yalov, S. I. *et al.* Synthesis of 2-aminothiazole derivatives. *Pharm. Chem. J.* **41,** 105–108 (2007).
16. Schuetz, A. *et al.* Structural basis of inhibition of the human NAD+-dependent deacetylase SIRT5 by suramin. *Structure* **15,** 377–389 (2007).
17. Pan, P. W. *et al.* Structure and biochemical functions of SIRT6. *J. Biol. Chem.* **286,** 14575–14587 (2011).
18. Davenport, A. M., Huber, F. M. & Hoelz, A. Structural and Functional Analysis of Human SIRT1. *J. Mol. Biol.* **426,** 526–541 (2014).
19. Jin, L. *et al.* Crystal structures of human SIRT3 displaying substrate-induced conformational changes. *J. Biol. Chem.* **284,** 24394–24405 (2009).
20. Trapp, J. *et al.* Adenosine mimetics as inhibitors of NAD+-dependent histone deacetylases, from kinase to sirtuin inhibition. *J. Med. Chem.* **49,** 7307–7316 (2006).
21. Outeiro, T. F. *et al.* Sirtuin 2 inhibitors rescue alpha-synuclein-mediated toxicity in models of Parkinson's disease. *Science* **317,** 516–519 (2007).
22. Tetko, I. V. *et al.* Virtual computational chemistry laboratory-design and description. *J. Comput.-Aided Mol. Des.* **19,** 453–463 (2005).
23. Sali, A. & Blundell, T. L. Comparative protein modelling by satisfaction of spatial restraints. *J. Mol. Biol.* **234,** 779–815 (1993).
24. Shen, M.-Y. & Sali, A. Statistical potential for assessment and prediction of protein structures. *Protein Sci.* **15,** 2507–2524 (2006).
25. Laskowski, R. A., MacArthur, M. W., Moss, D. S. & Thornton, J. M. PROCHECK: a program to check the stereochemical quality of protein structures. *J. Appl. Crystallogr.* **26,** 283–291 (1993).
26. Molecular Operating Environment (MOE), Version 2012.10, Chemical Computing Group Inc.: Montreal, QC, Canada, 2012.
27. Duan, Y. *et al.* A point□charge force field for molecular mechanics simulations of proteins based on condensed□phase quantum mechanical calculations. *J. Comput. Chem.* **24,** 1999–2012 (2003).
28. Jakalian, A. *et al.* Fast, efficient generation of high□quality atomic charges. AM1□

BCC model: II. Parameterization and validation. *J. Comput. Chem.* **23,** 1623–1641 (2002).

29. Wang, J., Wolf, R. M., Caldwell, J. W., Kollman, P. A. & Case, D. A. Development and testing of a general amber force field. *J. Comput. Chem.* **25,** 1157–1174 (2004).

30. Pang, Y.-P., Zinc Protein Simulations Using The Cationic Dummy Atom (CADA) Approach, http://www.mayo.edu/research/labs/computer-aided-molecular-design/projects/zinc-protein-simulationsusing-cationic-dummy-atom-cada-approach (2014).

31. Jorgensen, W. L., Chandrasekhar, J., Madura, J. D., Impey, R. W. & Klein, M. L. Comparison of simple potential functions for simulating liquid water. *J. Chem. Phys.* **79,** 926–935 (1983).

32. Pastor, R. W., Brooks, B. R. & Szabo, A. An analysis of the accuracy of Langevin and molecular dynamics algorithms. *Mol. Phys.* **65,** 1409–1419 (2006).

33. Darden, T., York, D. & Pedersen, L. Particle mesh Ewald: An N□log(N) method for Ewald sums in large systems. *J. Chem. Phys.* **98,** 10089–10092 (1993).

34. Ryckaert, J.-P., Ciccotti, G. & Berendsen, H. J. C. Numerical integration of the cartesian equations of motion of a system with constraints: molecular dynamics of n-alkanes. *J. Comput. Phys.* **23,** 327–341 (1977).

35. Glide, Schrödinger Suite v2012-5.8. Schrödinger Inc., New York, US, 2012.

36. Heltweg, B., Trapp, J. & Jung, M. In vitro assays for the determination of histone deacetylase activity. *Methods* **36,** 332–337 (2005).

37. Krishan, A. Rapid flow cytofluorometric analysis of mammalian cell cycle by propidium iodide staining. *J. Cell Biol.* **66,** 188–193 (1975).
